# Collagen hydroxylation couples NAD+/NADH dynamics to tumor dormancy and reactivation

**DOI:** 10.21203/rs.3.rs-6986228/v1

**Published:** 2025-07-10

**Authors:** Daniela De Martino, Begoña Zapatería, Jaclyn B. Dunne, Stanislav Drapela, Kailie Matteson, Duncan Oruko, Taylor Humphrey, Tyler Jonhston, Betsy Ann Varghese, Alessandra I. Riggio, Kanishka Tiwary, Erin Bresnahan, Jonathan Barra, Allison Sowa, William Jenssen, Simone Sidoli, Alana L. Welm, Margarida Barroso, Wayne Stallaert, Ana P. Gomes, Peggi M. Angel, Esperanza Arias, Jose Javier Bravo-Cordero

**Affiliations:** [1] The Tisch Cancer Institute, Icahn School of Medicine at Mount Sinai, New York, New York, USA.; [2] Department of Medicine and Department of Pathology at Albert Einstein College of Medicine, Bronx, New York, USA; [3] Department of Cell and Molecular Pharmacology and Experimental Therapeutics, Medical University of South Carolina, Charleston, South Carolina 29425, United States.; [4] Department of Molecular Oncology, Moffitt Cancer Center, Tampa, Florida, USA; [5] Department of Molecular and Cellular Physiology, Albany Medical College, Albany, NY, 12208, USA.; [6] Department of Computational and Systems Biology, UPMC Hillman Cancer Center, University of Pittsburgh, Pittsburgh, PA, USA.; [7] Department of Oncological Sciences, Huntsman Cancer Institute, University of Utah, Salt Lake City, UT 84112, USA.; [8] Department of Biochemistry Albert Einstein College of Medicine, Bronx, New York, USA; [9] The Montefiore Einstein Comprehensive Cancer Center and The Einstein Nathan Sock Aging Research Center at Albert Einstein College of Medicine, Bronx, New York, USA

## Abstract

Metastasis remains the leading cause of cancer-related mortality. Disseminated tumor cells (DTCs) colonize distant organs where they enter a prolonged state of quiescence, named cellular dormancy, within collagen-rich extracellular matrix (ECM) niches. How dormant cells regulate the formation of collagen-rich niches and the mechanisms maintaining collagen proteostasis during dormancy and reactivation are not understood. Here, we identify prolyl hydroxylase P4HA2 as a key regulator of tumor dormancy through its dual role in collagen proline hydroxylation and mitochondrial function. We demonstrate that P4HA2-mediated proline hydroxylation of collagens balances the NAD+/NADH ratio, sustaining dormancy by limiting mitochondrial activity. Loss of P4HA2 disrupts collagen proteostasis, induces autophagy, and activates the proline catabolism enzyme ALDH4A1, lowering the NAD+/NADH ratio, which fuels mitochondrial energetics and triggers DTC awakening. Notably, ALDH4A1 is essential for the survival of these reactivated dormant cells, and its depletion induces apoptosis upon awakening, revealing a metabolic vulnerability in reactivated dormant cells. Our findings establish a previously unrecognized link between collagen homeostasis, NADH metabolism and tumor cell dormancy, unveiling a mechanistic framework for identifying actionable targets to eliminate DTCs and prevent metastatic relapse.

## INTRODUCTION

Metastasis is a multiscale process in which cancer cells have to adapt to different tissue microenvironments to successfully establish metastatic lesions^1^. After intravasation into the bloodstream, DTCs must survive in circulation and extravasate to colonize the target organs^2,3^. Upon extravasation, DTCs can remain in a dormant, non-proliferative state for years, evading the immune system^4^ before they restore their growth and form metastatic lesions^5–9^. How DTCs succeed in colonizing metastatic organs and survive in this dormant state is not well understood and holds cues to prevent metastasis outgrowth. From a clinical perspective, the presence of dormant DTCs is a threat to cancer patients due to their ability to awake and form metastasis. Thus, targeting DTCs as a strategy to prevent metastasis is a therapeutic option that will prevent disease progression. However, the lack of understanding on the dynamics of DTC dormancy and reawakening limits strategies to prevent metastatic outbreaks and improve patient outcomes^8,10,11^.

Work from different laboratories has shown that the dormant state of DTCs is regulated by both intrinsic and extrinsic factors^9,12,13^. Among these factors, the extracellular matrix (ECM) plays a pivotal role in regulating tumor dormancy^13^. Dormant DTCs utilize cell-intrinsic ECM-related pathways to sustain their quiescence state^14,15^. These mechanisms include the production of collagens, such as COL17A1^16^ and COL3A1^15^, as well as other ECM-related proteins like TGFb2^17^ and osteopontin^18^. Collectively, these studies support the hypothesis that dormant cells assemble collagen-rich niches to sustain their quiescence state. However, the precise mechanisms regulating collagen proteostasis (the balance of collagen synthesis, modification and degradation^19^) required for effective niche construction and tight dormancy control remain poorly understood.

Collagen biosynthesis is a multistep process requiring extensive post-translational modifications (PTMs) including hydroxylation and glycosylation, which ensure proper collagen folding and fine-tune collagen proteostasis^20^. Proline residues are hydroxylated by three isoenzymes from the collagen prolyl-4 hydroxylase (C-P4H) family (P4HA1, P4HA2 and P4HA3) and by hydroxylases from the C-P3H family (LEPREL1, LEPREL2 and LEPRE). Prolyl 4-hydroxylation and 4-hydroxyproline content (4HYP) are critical for individual procollagen molecule stability, as they enable the formation of structurally stable triple helices at a physiological temperature^21,22^. Notably, in estrogen receptor-positive (ER+) breast tumors, P4HA1 was identified as part of a tumor cell dormancy signature that predicted dormancy and late relapse^23^. However, its role as well as the role of other prolyl hydroxylases in tumor dormancy remain to be investigated.

Disruption in collagen proteostasis (i.e. through collagen mutations or with inhibitors of P4H enzymes^24,25^) impacts the physiology of internal organelles, such as the endoplasmic reticulum (ER), triggering ER-stress and activating autophagy for clearance of misfolded collagens^26^. Autophagy is a catabolic process that degrades intracellular components within the lysosomal compartment, including -among others- proteins and organelles. Cells activate autophagy to maintain protein homeostasis, eliminate misfolded or damaged cytosolic components, and sustain energy balance by recycling breakdown products^27^. Additionally, autophagy regulates organelle homeostasis and reshapes the mitochondrial network through mitophagy^28,29^. Notably, autophagy plays a critical role in cancer progression^30^ and contributes to tumor dormancy^31,32^.

In this study, we identified P4HA2 as a central regulator of tumor dormancy, functioning at the interface of collagen proteostasis, autophagy and mitochondrial metabolism. Dormant cells fine-tune mitochondrial function by regulating intracellular collagen proteostasis through P4HA2, in an ALDH4A1-dependent manner, thus modulating NAD(H) levels to control dormancy. Our study reveals a new mechanism to control tumor cell dormancy that may be exploited to prevent the awakening or eliminate DTCs.

## RESULTS

### Collagen proline hydroxylation is essential for sustaining dormancy

**Figure 1.**
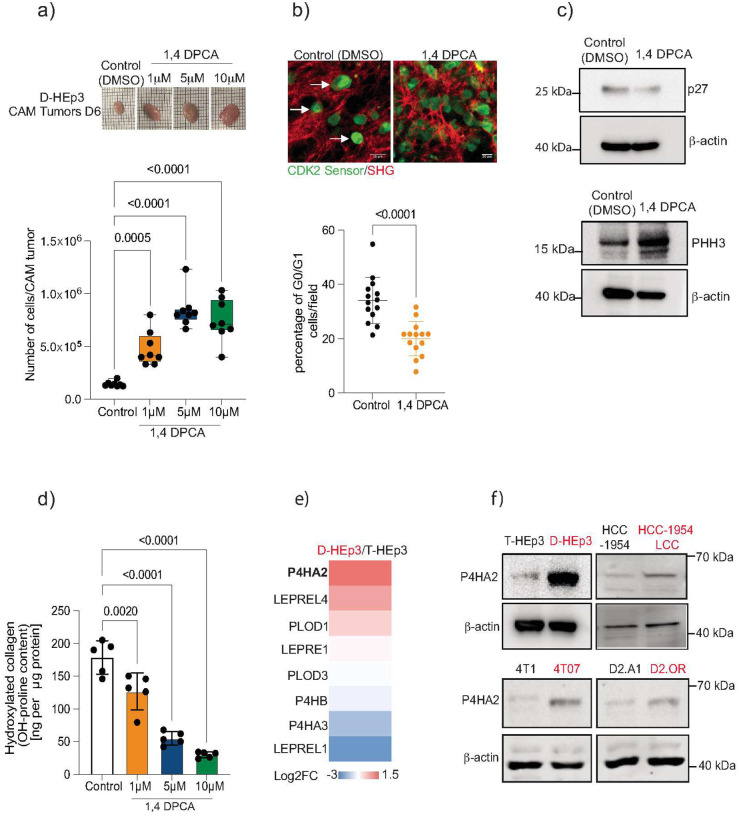
Collagen Proline hydroxylation is required for tumor cell dormancy A. Upper panel: Representative images of CAMs 6 days (D6) after inoculation of control DMSO and 1,4 DPCA-treated D-HEp3 cells. Lower panel: Number of cells per tumor in CAM assays (n= 8 CAMs per group). D-HEp3 cells were treated for 48 hours with 1,4 DPCA at different concentrations (1, 5, 10μM) and inoculated in fertilized chicken embryos. Statistical analysis was performed using one-way ANOVA followed by Dunnett’s test. B. Representative multiphoton images of control DMSO and 1,4 DPCA-treated D-HEp3 cells expressing a CDK2 sensor (green) in CAMs. Second harmonic generation is shown in red (SHG). Scale bar: 20μm. Quantification of the percentage of G0/G1 cells (with nuclear accumulation of CDK2 sensor) in D-HEp3 DMSO control (n= 6 CAMs, 14 fields) and D-HEp-3 treated with 1,4 DPCA at 5μM in CAMs (n=6 CAMs, 14 fields). Differences between groups were assessed using an unpaired two-tailed t-test. C. Western blot for p27, phospho-histone3 (PHH3) and β-actin in D-HEp3 control and treated with 5μM of 1,4 DPCA for 48 hours. D. Quantification of hydroxylated collagen levels in CAM tumors from D-HEp3 control cells (DMSO) and treated with different concentrations of 1,4 DPCA (1, 5, 10μM). Concentration of hydroxyproline is represented as ng of hydroxyproline per μg of total protein content (n=5 CAMs). Group comparisons were assessed using one-way ANOVA with Dunnett’s post-hoc test to identify differences relative to the control group. E. List of significantly dysregulated prolyl 4 hydroxylases (P4H) and prolyl 3 hydroxylases (P3H) genes in D-HEp3 nodules and T-HEp3 tumors grown in nude mice. Data are represented as log2 fold change D-HEp3/T-HEp3. F. Western blot analysis of P4HA2 expression in D-HEp3, 4T07, D2.0R and HCC1954 LCC (dormant) compared to T-HEp3, 4T1, D2.A1 and HCC1954 (proliferative) cells. b-actin was used as loading control.

To investigate the role of collagen proline hydroxylation in maintaining tumor cell dormancy we employed 1,4 DPCA, a high efficiency inhibitor of prolyl-4 hydroxylases^33,34^, in a well-established dormancy model from human head and neck squamous cell carcinoma^15,35^. When inoculated *in vivo*, dormant head and neck D-HEp3 cancer cells remain quiescent, forming small nodules in the chicken chorioallantoic membrane (CAM)^35^ or persisting as single DTCs or small clusters in the lungs of mice^15^. In contrast, the proliferative counterpart, T-HEp3 cells, rapidly grow into large tumors in CAMs and metastases in mouse lungs. Treatment with 1,4 DPCA disrupted dormancy, inducing proliferation in D-HEp3 cells *in vivo* (Fig. 1a). A similar effect was observed in the mouse mammary dormant tumor cell line D2.0R (**Fig. Supp. 1a**). Analysis of the quiescence status of D-HEp3 cells *in vivo* using a CDK2 biosensor^15,36^ showed a significant reduction in the number of cells in the G0/G1 phase following 1,4-DPCA treatment (Fig. 1b). Consistent with this, the dormancy marker p27 decreased, while the proliferation marker phospho-histone H3 (PHH3) increased, further confirming that 1,4-DPCA-treated D-HEp3 cells had exited their quiescent state (Fig. 1c). Importantly, 1,4-DPCA treatment significantly reduced total collagen hydroxylation levels *in vivo* (Fig. 1d). To further identify specific prolyl hydroxylases contributing to dormancy maintenance, we analyzed the gene expression profiles of C-P4H and C-P3H enzymes in dormant D-HEp3 versus proliferative T-HEp3 cells^15^. Among the differently expressed prolyl hydroxylases, P4HA2 emerged as the most significantly upregulated in dormant cells (Fig. 1e). This was further validated in western blot analyses and tumor tissue immunofluorescence, both of which confirmed that P4HA2 was expressed at higher levels in D-HEp3 than in T-HEp3 cells (Fig. 1f and **Supp. 1b**). To determine whether P4HA2 upregulation is a conserved feature of dormancy, we examined its expression in multiple breast cancer dormancy models, including murine D2.0R and 4T07 and human HCC1954-LCC. In all cell lines analyzed, P4HA2 expression was consistently increased in dormant cells compared to their proliferative counterparts (Fig. 1f). Conversely, R-HEp3 cells, which spontaneously escape dormancy *in vivo*, displayed reduced P4HA2 expression compared to D-HEp3 (**Fig. Supp. 1c**). Further supporting its relevance in clinical dormancy, high levels of P4HA2 were found in residual dormant cells in a HER2 therapy-associated mouse model when compared with the primary tumor^37^. Lastly, analysis of grossly uninvolved tissues (lung and liver) collected from the rapid autopsy of a HER2+ breast cancer patient revealed that DTCs are positive for P4HA2 (**Fig. Supp. 1d**). Altogether, these results highlight P4HA2 as a conserved marker of the dormant state in cancer cells.

### P4HA2 is functionally linked to dormancy.

**Figure 2.**
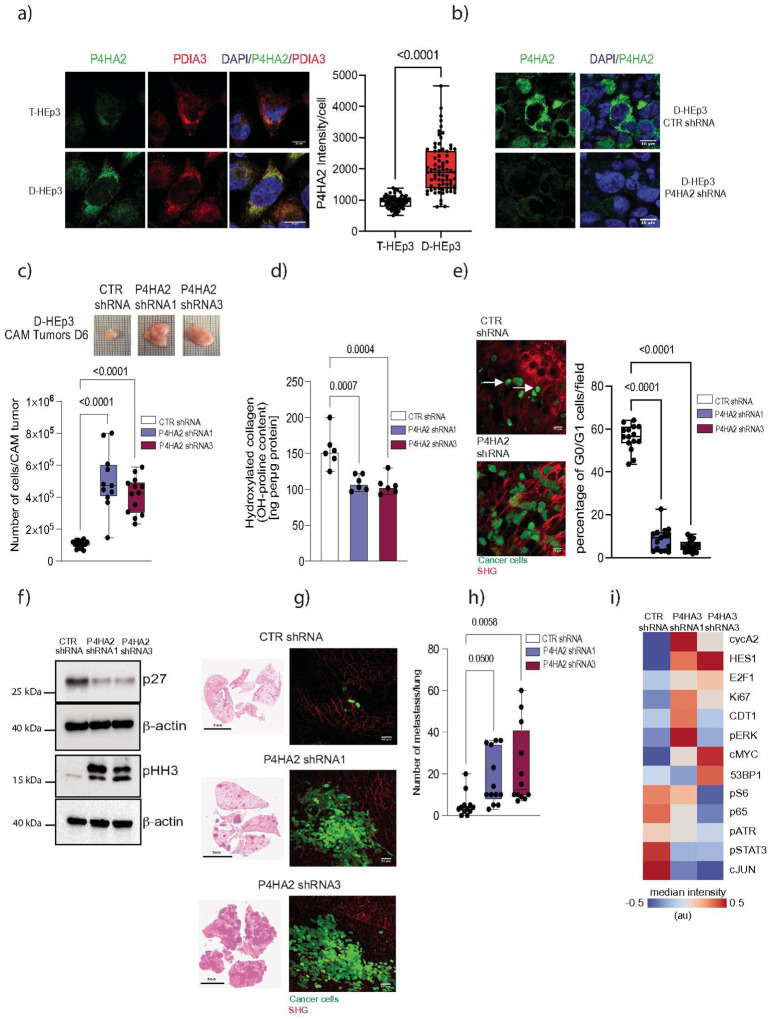
P4HA2 is required for tumor cell dormancy A. Representative immunofluorescence images of T-HEp3 and D-HEp3 cells stained for P4HA2 (green), ER marker PDIA3 (red) and DAPI (blue) for nuclei staining. Scale bar: 10μm.The graph shows quantification of P4HA2 intensity for D-HEp3 cells compared to T-HEp3 cells (n=74 cells/group). The two groups were statistically compared with the Mann-Whitney test. B. Immunofluorescence images of D-HEp3 control shRNA nodules and P4HA2 KD tumors from CAMs stained for P4HA2 (green). DAPI was used for nuclei counterstaining. Scale bar: 10μm C. Representative images of D-HEp-3 control nodules and P4HA2 KD tumors from CAMs. Number of cells from D-HEp3 control nodules (n=19 CAMs) and D-Hep3 P4HA2 knockdown tumors (shRNA1 n=11 and shRNA2 n=13 CAMs) are represented. To assess group differences, one-way ANOVA was conducted, and Dunnett’s test was applied to compare each group to the control. D. Quantification of hydroxylated collagen levels in P4HA2 KD CAM tumors (n=6 CAMs) compared with D-HEp3 control nodules (n=6 CAMs). Statistical significance is assessed by one-way ANOVA followed by Dunnett’s test to compare treatments to the control. E. Representative images of D-HEp3 shRNA control nodules and D-HEp3 P4HA2 KD cells expressing a CDK2 biosensor (green). Second harmonic generation is shown in red (SHG). Scale bar: 20μm. Quantification of the percentage of G0/G1 cells per field in D-HEp3 cells shRNA control (n=15 fields, 3 CAM) and D-HEp3 P4HA2 KD (P4HA2 shRNA1 n=15 and P4HA2 shRNA3 n=15 fields, 6 CAM total) Differences between groups were analyzed by one-way ANOVA followed by Dunnett’s test to compare treatments to the control. F. Western blot for p27 protein and PHH3 levels in P4HA2 KD D-HEp3 compared with D-HEp3 control. b-actin was used as loading control. G. Left panels: Representative images of hematoxylin/eosin staining of mouse lungs derived from tail vein injections of D-HEp3 shRNA control and D-HEp3 P4HA2 KD. Scale bar: 5mm. Right Panels: Representative multiphoton images of solitary cells in D-HEp3 shRNA control and D-HEp3 P4HA2 KD micrometastasis in the lungs. In green, cancer cells expressing GFP. SHG signal (in red) highlights fibrillar collagens in the extracellular matrix Scale bar: 50μm. H. Graph representing number of metastases per lung/mouse in D-HEp3 P4HA2 KD group compared with D-HEp3 control group. Statistical analysis included one-way ANOVA followed by Dunnett’s test to compare all group to the control. (shRNA control n=12, shRNA1= 13, shRNA2=12 mice) I. Heat map showing protein levels measured by immunofluorescence in control and P4HA2-depleted primary tumors in mice. n>10000 cells per condition from 3 different tumors.

P4HA2 localizes to the endoplasmic reticulum (ER) in dormant D-HEp3 cells (Fig. 2 a; **Fig. Supp 1b**). To explore the functional role of P4HA2 in dormancy, we generated knockdown (KD) models in D-HEp3 and D2.0R cells (**Fig. Supp. 2 a,b,c**). P4HA2 KD disrupted dormancy and restored proliferation *in vivo* (Fig. 2c, **Fig. Supp 2d,h**). Depletion of P4HA2 led to a significant reduction in collagen proline hydroxylation levels, an increase in PHH3, a decrease in p27 and a reduced number of G0/G1-phase cells, confirming an escape from dormancy (Fig. 2d–f).

In mice, tumors formed from P4HA2-depleted D-HEp3 cells exhibited higher levels of PHH3, an increased tumor volume and a shorter latency period when compared with control (**Fig. Supp. 2e,f,g; Fig. Supp. 3a,b**). Furthermore, *in vivo* tail vein injection experiments, P4HA2-deficient D-HEp3 cells generated a higher number of lung metastases, whereas control cells persisted as solitary cells or small clusters (Fig. 2g, h). Similar results were obtained in a D2.0R tail vein model where mice inoculated with P4HA2 KD cells exibit an higher number of metastatic cell clusters in the lung (**Fig. Supp. 2i**). To further dissect the signaling network underlying awakening, we employed multiplex single-cell imaging to profile cell cycle-related protein expression *in vivo*. Comparing of single-cell profiles between control nodules and P4HA2-depleted D-HEp3 tumors, revealed distinct differences in key cell cycle regulators. P4HA2 depletion resulted in increased expression of proliferative markers (cycA2, E2F1, Ki67, CDT1, cMYC, pERK) highlighting a shift in the cell cycle signaling network that drives awakening of P4HA2 dormant cells (Fig. 2i). Finally, to assess whether P4HA2 is sufficient to induce dormancy, we overexpress human P4HA2 in T-HEp3 cells. P4HA2 overexpressing T-HEp3 form smaller tumors when compared with control T-Hep3 ones (**Fig. Supp. 2j,k**). Together, these findings establish P4HA2 as a critical regulator of tumor cell dormancy, linking its expression to quiescence and its depletion to the reactivation of proliferation and metastatic outgrowth via modulation’of cell cycle regulatory networks.

### P4HA2 activity in dormant cells regulates the proline hydroxylation landscape of fibrillar collagens.

**Figure 3.**
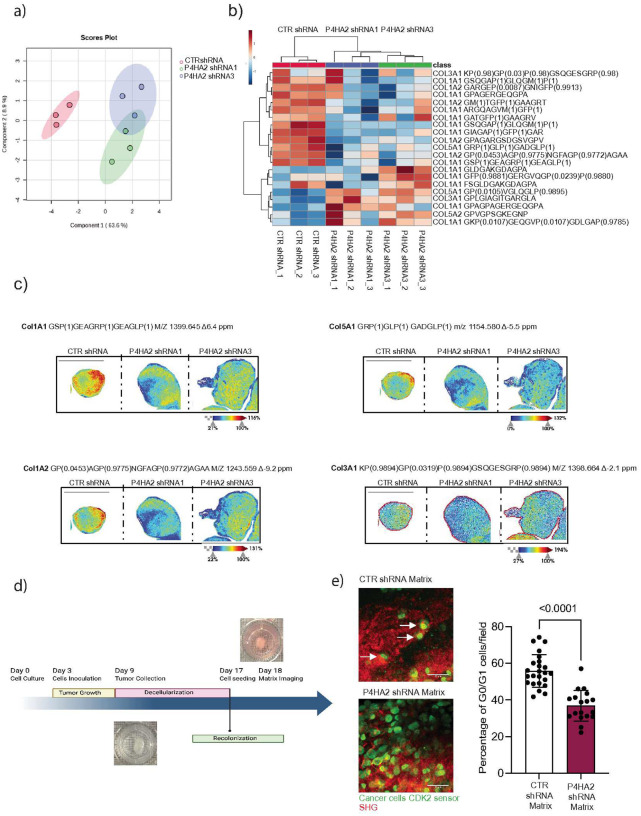
P4HA2 regulates the spatial distribution and abundance of proline modified collagens A. Principal component analysis of human collagen peptides from control and P4HA2-depleted D-HEp3 tumors. n=3 tumors per condition. B. Human collagen peptides showing significant differences between control and P4HA2-depleted D-HEp3 tumors. C. Representative images showing single HYP modified human collagens peptides with differential expression patterns between control and P4HA2 depleted D-HEp3 tumors. D. Schematic representation of the experimental plan for ECM decellularization and reseeding experiment. E. Representative ex vivo multiphoton images of CDK2 sensor (green) localization in D-HEp-3 parental cells repopulating D-HEp3 shRNA Control or D-HEp3 P4HA2 KD-derived ECM matrices. Scale bar: 50μm. The graph shows the percentage of G0/G1 cells per field. G0/G1 cells were analyzed in 6 different field per tumor.(CTR shRNA matrix: 4 CAMs, P4HA2 shRNA3 matrix: 3). Differences between groups were assessed using an unpaired two-tailed t-test.

To identify the specific collagens and their associated proline hydroxylation modifications regulated by P4HA2, high-resolution accurate mass (HRAM) LC-MS/MS proteomics and collagen mass spectrometry imaging (MSI) were performed on control and P4HA2-depleted D-HEp3 awakened cells *in vivo*. Principal components analysis using human collagen peaks showed no overlap between control and P4HA2-depleted D-HEp3 tumors, revealing differences in ECM collagen composition upon P4HA2 depletion (Figure 3a and **Fig. Supp. 3c,d,e**). Peptide abundance analysis further revealed specific collagen peptides impacted by P4HA2 depletion in each of the collagens analyzed (Fig. 3b).

To investigate the spatial distribution of collagen hydroxyproline (HYP) modifications, collagen MSI was emplyed to map HYP collagen peptides within the tumor tissue. The spatial localization of HYP peptides for COL1A1, COL1A2, COL5A1 and COL3A1 exhibited distinct region-specific patterns in dormant D-HEp3 compared to awakened P4HA2-depleted D-HEp3 cells (Fig. 3c), suggesting differential regulation of collagen hydroxylation across tumor zones. For example, in COL1A1, COL1A2, COL3A1 and COL5A1 HYP peptides were enriched in the tumor nodule boundaries, a pattern that was lost in P4HA2-depleted tumors (Fig. 3c). These findings suggest that collagen hydroxylation patterns shift during dormancy and awakening, with the spatial distribution of HYP peptides potentially contributing to establishing the ECM niche that sustains dormant cells.

To determine the impact of 4HYP-modified collagen matrix produced by P4HA2-depleted cells on tumor cell quiescence, we generated decellularized matrix scaffolds from D-HEp3 nodules or P4HA2-depleted D-HEp3 awakened tumors and seeded D-HEp3 cells expressing a CDK2 biosensor onto them (Fig. 3d). We found that D-HEp3 cells seeded on matrices derived from P4HA2-depleted awaken tumors have decreased number of cells in G0/G1 state, suggesting the escape from dormancy (Fig. 3e). These findings indicate that altered collagen proline hydroxylation can impact tumor cell quiescence.

### Autophagy is regulated by P4HA2 in dormant cancer cells.

**Figure 4.**
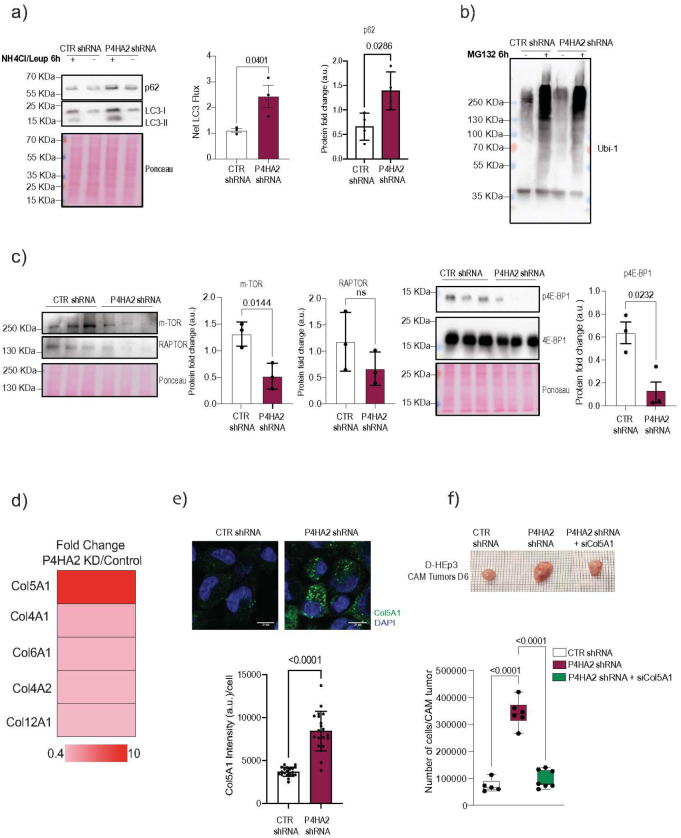
P4HA2 modulates autophagy. A. Immunoblot analysis of LC3 and p62 levels in control and P4HA2 KD D-HEp3 cells upon treatment with ammonium chloride (20 μM) and leupeptin (100 μM) for 6 hours. Ponceau staining was used as protein loading control. Quantification of autophagic flux calculated as LC3 Net flux relative to D-HEp3 shRNA control and D-HEp3 P4HA2 KD cells is shown. Quantification of p62 levels in D-HEp3 shRNA control in comparison with D-HEp3 P4HA2 KD cells. Statistical analysis was performed using an unpaired two-tailed t-test. B. Representative immunoblot of ubiquitinated proteins in control and P4HA2 KD D-HEp3 cells following proteasome inhibition with MG132 (5μM) treatment for 6 hours. C. Western blot and proteins levels quantification of key components of the mTOR signaling pathway, including mTOR, RAPTOR, phosphorylated 4E-BP1, and total 4E-BP1, in control and P4HA2 knockdown D-HEp3 cells. Ponceau staining is provided as a loading control. Statistical analysis was performed using an unpaired two-tailed t-test. D. Heatmap representation of fold change in collagens accumulation upon ammonium chloride/leupeptin treatment in P4HA2 knockdown D-HEp3 cells relative to control D-HEp3 cells. E. Representative images of COL5A1 and DAPI staining in control and P4HA2 KD cells. Quantification of COL5A1 intensity per cell is shown (n= 23 cells per condition). Scale bar: 10 μm. F. Representative images of CAMs tumors derived from D-HEp3 cells control, P4HA2 knockdown, and P4HA2 knockdown cells transiently transfected with a small interfering RNA targeting COL5A1. The graph shows the number of cells per tumor per each group. Differences between groups were analyzed by one-way ANOVA followed by Dunnett’s test to compare treatments to the control.

Misassembled collagens induce endoplasmic reticulum (ER) stress and can be targeted for degradation in the lysosomal compartment through autophagy^26^. In P4HA2-depleted D-HEp3 cells, we observed increased levels of p-PERK^Thr 982^, indicating an activation of the ER stress pathway (**Fig. Supp. 4a**). Additionally, ER expansion, previously reported upon disruption of collagen folding^25^, was also evident in P4HA2-depleted D-HEp3 cells (**Fig. Supp. 4b**). Based on these observations, we hypothesized that ER stress upon P4HA2 depletion may trigger autophagy to degrade misfolded collagens as a protective strategy against collagen accumulation. To test this hypothesis, we measure LC3 flux, a well established marker of autophagy, in control and P4HA2-depleted D-HEp3 cells (Fig. 4a). Treatment with lysosomal inhibitors (NH4Cl/Leup; ammonium chloride and leupeptin) to block lysosomal degradation^38^, confirmed a significant increase in LC3-II flux and in another widely used autophagy marker, SQSTM1 also known as p62, in P4HA2-depleted cells when compared with controls D-HEp3 cells (Fig. 4a). Accumulation of misfolded proteins was induced by the addition of the proteasome inhibitor MG132 causing an increase of ubiquitinated proteins; similar intensity indicates no major changes in the proteosome system between both control and P4HA2 -depleted D-HEP3 cells (Fig. 4b). mTOR is a well characterized autophagy inhibitor and a known substrate of P4HA2^39^. P4HA2 depletion was associated with decreased mTOR protein, RAPTOR (key regulatory protein that associates with mTOR to form the mTORC1 complex) and 4E-BP1 phosphorylation, a main target of mTORC1 activity, supporting the activation of autophagy (Fig. 4c), as reflected by accumulation of p62 and LC3 in P4HA2 - depleted D-HEP3 cells (Fig. 4a).

To determine whether collagens are specifically targeted for autophagic degradation upon P4HA2 depletion, we conducted proteomic analysis in control and P4HA2-depleted D-HEp3 cells in the presence or absence of the aforementioned lysosomal inhibitors (NH4Cl/Leup). The accumulation of proteins upon lysosomal inhibition suggests their routing for lysosomal degradation via autophagy. We found that COL5A1, COL4A1, COL4A2, COL6A1 and COL12A1 accumulated in P4HA2-depleted cells (Fig. 4d and **Fig. Supp. 4c**). Among these, COL5A1 exhibited the highest accumulation in P4HA2-depleted cells and localize within lysosomes when compared to control cells (Fig. 4e and **Supp. 4d**). Interestingly, COL5A1 did not accumulate when proteasome activity was inhibited, reinforcing the idea that its degradation mainly relies in the autophagic system (**Fig. Supp. 4e**). *In vivo* experiments demonstrated that COL5A1 depletion (Fig. 4f and **Fig. Supp. 4f**) prevented the awakening of P4HA2 KD D-HEp3 cells, suggesting a role for COL5A1 in the reactivation of P4HA2-depleted dormant cells. Altogether, these findings indicate that P4HA2 regulates autophagy activity to control the proteostasis of COL5A1, ultimately influencing the dormant state of cancer cells.

### P4HA2 regulates mitochondrial topology and function.

**Figure 5.**
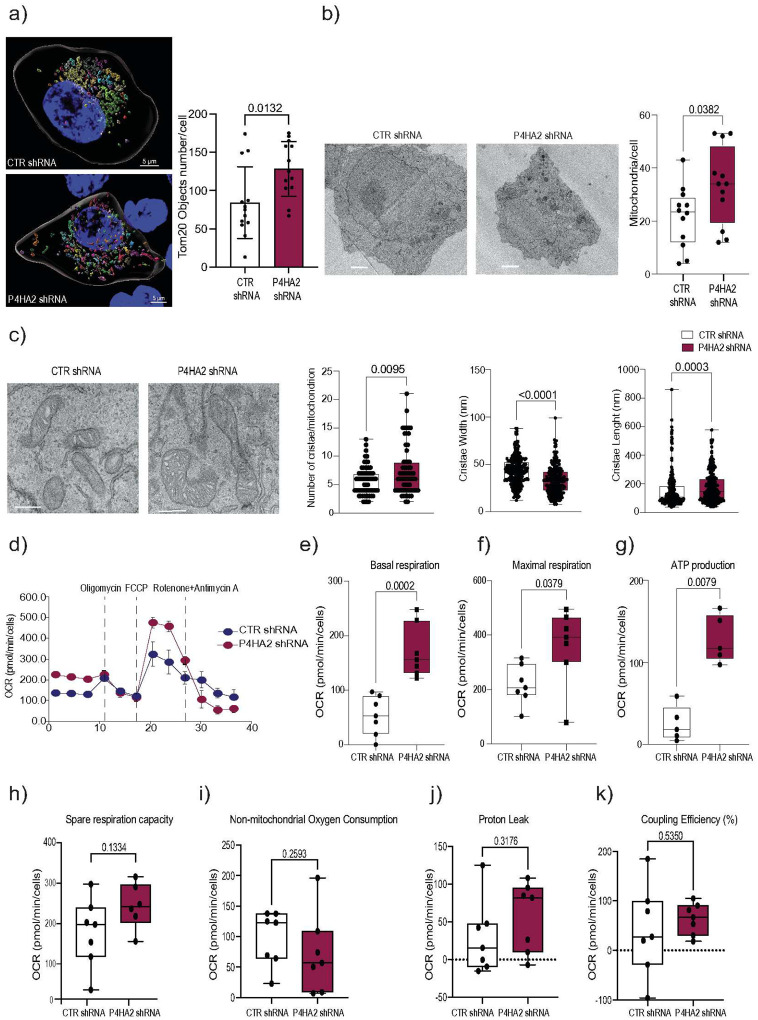
P4HA2 activity regulates mitochondria architecture and respiration. A. 3D rendering of Tom20 staining and quantification of mitochondrial object number in cells expressing control shRNA (CTR shRNA) and P4HA2 shRNA (object ID color coded). Mitochondria were segmented from 3D confocal image stacks and object numbers were quantified. Scale bar: 5μm. Statistical analysis was performed using unpaired two-tailed t-tests. B. Representative electron microscopy images of D-HEp3 KD control cells (1200x magnification, scale bar: 2 μm) and D-HEp3 P4HA2 KD cells (1200x magnification, scale bar: 2 μm). Right panel: The graph show the number of mitochondria per cell in D-HEp3 shRNA control cells versus D-HEp-3 P4HA2 knockdown cells. (n=12 cells per group). Statistical analysis was performed using an unpaired two-tailed t-test. C. Representative electron microscopy images of mitochondria structural details from D-HEp3 control (scale bar: 500nm) and D-HEp3 P4HA2 KD (scale bar: 400nm) cells.The graphs show the difference in the number of cristae, cristae width (in nm) and length (in nm) per mitochondrion between D-HEp3 shRNA Control cells (n=51 mitochondria, 231 cristae analyzed cells) and D-HEp3 P4HA2 knockdown cells (n=52 mitochondria, 310 cristae analyzed cells). Differences between groups were analyzed by one-way ANOVA followed by Dunnett’s test to compare treatments to the control. D: Real-time measurement of oxygen consumption rate (OCR, pmol/min/cell) using the Seahorse XF Analyzer in control and P4HA2-depleted cells. Mitochondrial respiration was assessed by sequential injection of oligomycin (1 μM), FCCP (1.5 μM), and rotenone/antimycin A (0.5 μM each), as indicated. Data represent mean ± SEM of 7 technical replicates and are representative of two independent experiment. E-K: Measurement of cellular basal respiration, maximal mitochondrial respiration rate, ATP production, spare respiratory capacity, Non-mitochondrial oxygen consumption, proton leak, and Coupling Efficiency expressed as oxygen consumption rate (pmol/min/cells) in D-HEp3 control cells compared to P4HA2 KD cells. A one-way ANOVA was performed to evaluate differences among groups, with Dunnett’s test used post hoc to compare each treatment group to the control group.

Changes in ER and autophagy function can impact mitochondrial activity^40^. To further characterize mitochondrial dynamics, we performed artificial intelligence-driven spatial analysis of mitochondria topology^41^ in dormant and P4HA2-depleted D-HEp3 cells (**Fig. Supp. 5a**). Our results demonstrate that dormant and proliferative cellular states can be discriminated with extreme accuracy based on mitochondrial topology (a set of 18 parameters based on the distance between organelle objects that can be divided into 6 nucleus-related (NPG) and 12 distance-related parameters (DPG)^41^) (**Fig. Supp. 5c**). In contrast, classification based on morphology (a group of 16 parameters describing organelle size and shape) yielded lower accuracy (**Fig. Supp. 5d**). Confusion matrix and t-SNE plots illustrate classification of these cellular states using the Random Forest machine learning algorithm. P4HA2-depleted D-HEp3 cells exhibited increased number of mitochondria with smaller average volumes, suggesting that P4HA2 KD promotes mitochondria fragmentation and reduced tubulation (Fig. 5a,b and **Fig. Supp. 5a, b**). These results indicate that P4HA2-depletion in D-HEp3 cells induces a significant alteration of mitochondrial distribution and morphology. Consistent with the observed increase in autophagy, P4HA2-depleted D-HEp3 cells also displayed enhanced mitophagy as seen with Keima mitophagy biosensor^42^, likely serving to efficiently remove damaged mitochondria while maintaining an active mitochondrial pool (**Fig. Supp. 5 e,f**). At the ultrastructural level, electron microscopy analyses revealed a higher number of cristae per mitochondrion in P4HA2-depleted D-HEp3 cells (Fig. 5c). Given these mitochondrial alterations, we assessed the impact of P4HA2 depletion on mitochondrial bioenergetics using the Seahorse Mito Stress Test assay in live cells. Loss of P4HA2 in D-HEp3 cells led to an increase in both basal and maximal mitochondrial respiration as well as to elevated ATP production (Fig. 5d–g), without affecting coupling efficiency as well as spare respiratory capacity (Fig. 5h–k). These findings suggest that P4HA2 plays a critical role in regulating mitochondrial oxidative phosphorylation capacity in dormant cancer cells.

### NAD+/NADH ratio is linked to dormancy and reactivation

**Figure 6.**
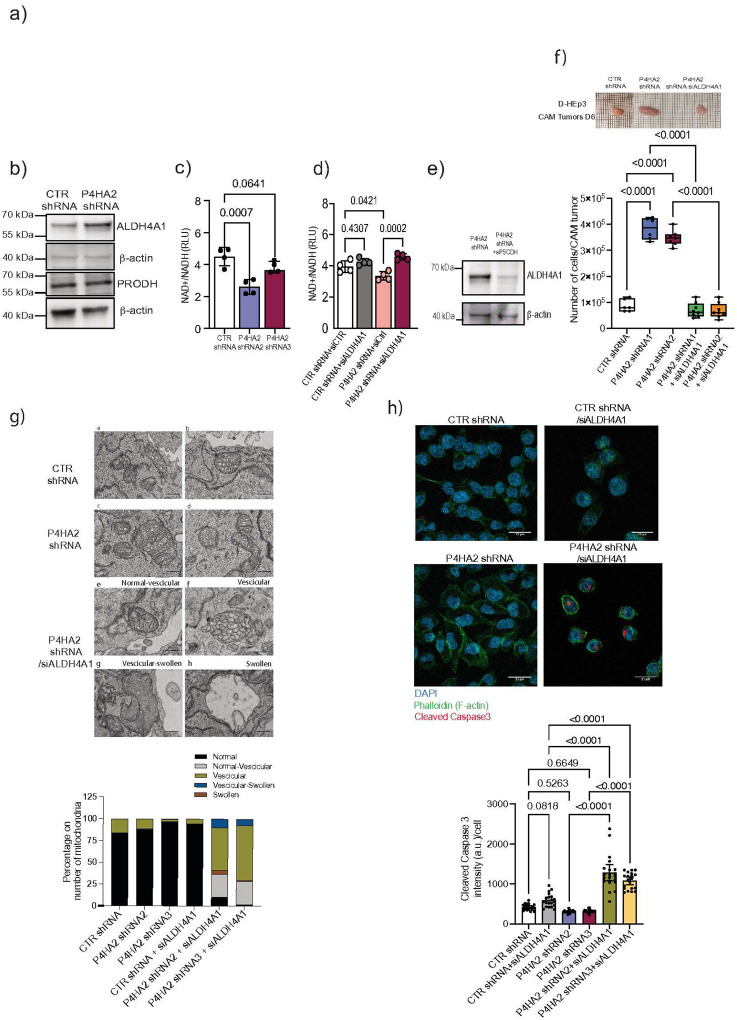
NAD+/NADH ratio is regulated by P4HA2 activity via ALDH4A1 A. Cartoon Representing Mitochondrial Proline Metabolism: The diagram illustrates the key enzymatic steps in mitochondrial proline metabolism, including proline oxidation by proline dehydrogenase (PRODH) and conversion of P5C to glutamate by aldehyde dehydrogenase 4A1 (ALDH4A1), with associated production of NADH and ATP. These reactions support the tricarboxylic acid (TCA) cycle and contribute to cellular energy production. B. Western blot analysis of ALDH4A1 (P5CDH) and PRODH protein levels in D-HEp3 shRNA control (CTR shRNA) and P4HA2 knockdown (P4HA2 shRNA) cells. β-actin was used as a loading control. C. NAD+/NADH ratio measured in control shRNA (CTR) and P4HA2 KD D-HEp3 cells (RLU: relative luminescence units). Statistical analysis was performed using an unpaired two-tailed t-test. (n=4 measurements per group were performed). D. NAD+/NADH ratio measured in CTR shRNA and P4HA2 knockdown cells (RLU: relative luminescence units), transfected with P5CDH or control siRNA. One-way ANOVA followed by multiple comparisons test was used to assess differences between experimental groups. (n=4 measurements per group were performed). E. Western blot analysis of ALDH4A1 protein levels in P4HA2 KD and P4HA2 KD D-HEp3 cells transiently transfected for 48hrs with P5CDH (ALDH4A1) siRNA. β-actin was used as loading control F. Upper panel: Representative images of CAM nodules and tumors derived from DHEp3 shRNA control, D-HEp3 P4HA2 shRNA2 and D-HEp3 P4HA2 shRNA2 + siP5CDH. Lower panel: Number of cells per CAM assay (n=6 of Control, n=6 P4HA2 shRNA1, n=7 P4HA2 shRNA2, n=8 P4HA2 shRNA1+ siP5CDH, n=8 P4HA2 shRNA2+ siP5CDH CAMs). Tumors were harvested 6 days after inoculation and cells were counted. A one-way ANOVA was performed to evaluate differences among groups, with Dunnett’s test used post hoc to compare each treatment group to the control group. G. Representative transmission electron microscopy (TEM) images of mitochondria ultrastructure (7000X) in D-HEp3 shRNA control and D-HEp-3 P4HA2 KD cells with or without silencing P5CDH (ALDH4A1). Scale bar: 500nm. The quantification categorizes mitochondria as normal, vesicular, vesicular-swollen or swollen based on morphological features and compared between different conditions. The analysis was carried out on 17 cells per each group. H. Representative immunofluorescence images showing cleaved caspase-3 (red), F-actin (green), and DAPI for nuclei (blue) in D-HEp3 shRNA control and P4HA2 KD cells with or without silencing P5CDH (ALDH4A1). The bar graph quantifies cleaved caspase-3 staining intensity per cell (20 cells per each group). Scale bar: 25 μm. One-way ANOVA followed by multiple comparisons test was used to assess differences between experimental groups.

Collagens serve as proline reservoirs, with approximately 20% of their composition consisting of proline residues^43^. Collagen degradation can release proline, which can be utilized as an energy substrate in the mitochondria, fueling metabolism through the TCA cycle^44^ (Fig. 6a). Based on our findings, we hypothesized that collagen degradation induced by P4HA2 depletion increases proline availability in dormant cells, which in turn trigger their awakening. Since proline is catabolized in the mitochondria by proline dehydrogenase (PRODH) and aldehyde dehydrogenase 4 family member A1 (ALDH4A1), we investigated the levels of both enzymes in P4HA2-depleted cells. Levels of ALDH4A1 (gene name *P5CDH*), yet not PRODH, were increased in P4HA2-depleted D-HEp3 cells compared to D-HEp3 control cells (Fig.6b). ALDH4A1 activity generates NADH through proline catabolism, which fuels the electron transport chain to produce ATP (Fig. 6a). Thus, we hypothesized that ALDH4A1 activity may contribute to the awakening of P4HA2-depleted cells by modulating the NAD+/NADH ratio, a central pillar of redox homeostasis and tightly linked to mitochondrial oxidative phosphorylation capacity. Our results indicate that P4HA2-depleted cells have a lower NAD+/NADH ratio than control dormant D-HEp3 cells and increased levels of L-glutamine and succinate, an intermediate metabolites of the TCA cycle (Fig. 6c, **Fig. Supp 6a-b**). While ALDH4A1 depletion in dormant D-HEp3 cells had no significant effect on NAD+/NADH ratio, that ratio was restored in P4HA2-depleted cells upon ALDH4A1 KD, confirming its role in regulating NAD+/NADH in P4HA2 KD awakened dormant cells (Fig. 6d). Further comparison of D-HEp3, T-HEp3 and R-HEp3 cells showed that proliferative cells with low P4HA2 levels (i.e.THEp3, R-HEp3) have similar NAD+/NADH ratios as P4HA2-depleted cells (**Fig. Supp. 6c**). Interestingly, supplementation of proline to dormant D-HEp3 cells, but not in T-HEp3 cells, disrupted NAD(H) balance, suggesting that dormant cells are highly sensitive to proline levels and adjust their NAD+/NADH ratio in response to proline availability (**Fig. Supp. 6d**). Notably, proline supplementation triggered the awakening of dormant D-HEp3 cells *in vivo* in an ALDH4A1-dependent manner and led to metastasis formation in mice (**Fig. Supp. 6e, f**).

To confirm whether ALDH4A1 depletion affects the awakening of P4HA2-depleted cells, we downregulated ALDH4A1 and observed that its depletion abrogated the awakening of these dormant cells (Fig. 6f). Given the altered mitochondria morphology in P4HA2-depleted cells, we further investigated the impact of ALDH4A1 depletion on mitochondria ultrastructure. Electron microscopy analysis revealed that upon ALDH4A1 depletion the mitochondria in P4HA2-depleted D-HEp3 cells exhibited increased vesicular and swollen structures^45^, indicative of mitochondrial damage (Fig. 6g). These results led us to hypothesize that the impaired awakening of P4HA2-deleted cells following ALDH4A1 downregualation may be due to an increased cell death. Consistent with this, cleaved caspase 3 staining revealed an higher number of apoptotic cells upon ALDH4A1 depletion, suggesting that loss of ALDH4A1 activity compromises the survival of awaken D-HEp3 cells by promoting apoptosis (Fig. 6h).

## DISCUSSION

**Figure 7.**
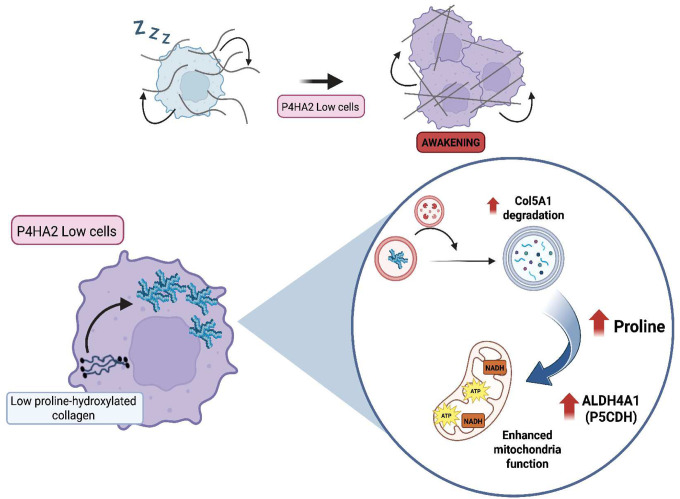
Proposed model for P4HA2 regulation of tumor dormancy. In dormant tumor cells, P4HA2 activity facilitates proper collagen folding by promoting proline hydroxylation. When P4HA2 is depleted (P4HA2 low), reduced proline hydroxylation leads to the accumulation of misfolded collagens. This triggers autophagy, which degrades the accumulated collagens and releases free proline. The recycled proline is then funneled into the mitochondria, where it is used as an energy source to support cellular awakening from dormancy.

In this study, we identify the collagen proline hydroxylase P4HA2 as a critical regulator of dormancy, through its role in maintaining NAD(H) balance. Our findings establish the NAD+/NADH ratio as a distinct metabolic hallmark that differentiates dormant from proliferative cancer cells. We demonstrate that inhibiting P4HA2 disrupts dormancy through a mechanism involving the autophagic degradation of intracellular collagens, the activation of proline catabolism via ALDH4A1 and the alteration of the NAD+/NADH ratio (Fig. 7). Our study reveals a tumor cell-intrinsic dormancy mechanism in which intracellular collagen proteostasis regulates the metabolic state of dormant cells by limiting collagen-derived proline availability. Overall, these findings highlights a previously unrecognized role of tumor-derived collagen as a key metabolic source of proline that fuels mitochondrial activity and governs dormancy.

The ECM of primary tumors and metastases consists of proteins derived from both tumor cells and the stromal compartments. While serving as a structural scaffold and signaling hub, the ECM also serves as a nutrient reservoir. In pancreatic ductal adenocarcinoma (PDAC)^46^, tumor cells scavenge collagen fragments from the tumor microenvironment to sustain their growth. Moreover, in the case of disseminated tumor cells, ECM remodeling plays a pivotal role in both tumor progression and the maintenance of the dormant state of DTCs, by creating collagen-rich niches that support the dormant state^15^. Our study expands this concept by uncovering a previously unrecognized function of intracellular collagens, such as COL5A1, which fuels mitochondrial activity through altered proline hydroxylation levels, linking collagen metabolism directly to dormancy regulation.

Previous studies in breast and prostate tumors revealed zonal regulation of collagens and their HYP-modified species^47,48^. Consistent with these findings, our spatial collagen proteomics analysis revealed spatial zonation of HYP-modified collagens in dormant cells *in vivo*, with their accumulation at discrete regions within dormant nodules. While the functional consequences of HYP-rich zones on tumor-stromal interactions remain unclear, we speculate that these microenvironments define specific dormancy-supportive niches that may also influence tumor-stroma interactions. For example, matrix architecture has been shown to regulate T cells infiltration in lung tumors, raising the possibility that HYP-rich ECM niches modulate tumor-immune interactions to either sustain dormancy or promote immune evasion^49^. Additionally, our study detected intracellular accumulation of specific collagens, suggesting that other collagens affected by P4HA2 depletion, such as COL3A1 and COL1A1, may be secreted in a misfolded form due to low HYP content. These collagens could disrupt matrix assembly^50^ and impact tumor cell signaling, as we showed in this study.

Early work by Ranganathan et al. identified the collagen chaperone Hsp47 as a downstream target of p38 signaling upregulated in dormant cells^51^. Hsp47 is required for proper procollagen folding^52,53^, and its loss in HSP47-deficient (HSP47−/−) fibroblasts, triggers autophagy to clear misfolded collagens, a process similar to what we observed upon P4HA2 depletion^26^. Autophagy activation facilitates the degradation of misfolded proteins, including collagens, generating essential building blocks for protein synthesis to sustain tumor growth^38^. P4HA2 has been shown to activate mTOR via hydroxylation^39^. Given that mTOR inhibits autophagy^54^, we propose that, in dormant cells, P4HA2 regulates dormancy not only through collagen metabolism but also via mTORC1 signaling. Collectively, these findings suggest a broader mechanism by which collagen homeostasis, governed by P4HA2 and presumably other proteins such as HSP47, contributes to dormancy niche formation.

A link between the ECM and mitochondria was previously proposed, although not in the context of cancer dormancy or metastasis. Early work by Zena Werb showed that integrins regulate mitochondrial function^55^, and more recently, muscle tissue analysis of COL6A1-deficient mice revealed mitochondrial ultrastructural defects, characterized by abnormal cristae, presumably due to disrupted integrin engagement^56^. Our study extends this concept to cancer by uncovering a novel role for intracellular collagens in regulating mitochondria ultrastructure. Dormant-to-awakening transitions were associated with increased cristae formation, respiration, and ATP production. Moreover, work by the Malladi lab showed that mitochondrial plasticity support the survival of dormant cells^57^ further reinforcing mitochondrial dynamics as an important aspect of tumor dormancy. While the precise molecular mechanisms by which P4HA2 influences mitochondrial architecture remain to be defined, we speculate that changes in proteins involved in cristae remodeling, such as OPA1 or DRP1, may mediate these mitochondrial changes^58–60^.

Nutrient availability plays a crucial role in shaping the metabolic state of tumors, yet the nutritional requirements of dormant cells remain poorly understood^7^. Proline supplementation has been shown to induce growth in breast tumor cells in mice^61^ and enhance mitochondrial function in senescent fibroblasts^62^. Interestingly, we demonstrate here that proline supplementation in dormant cells decrease the NAD+/NADH ratio and breaks their dormancy *in vivo* in an ALDH4A1-dependent manner, mirroring the metabolic phenotype observed upon P4HA2 depletion. This result highlights nutrient composition within the tissue microenvironment as a potential source of dormancy and awakening signals, suggesting that changes in nutrient availability may trigger the reactivation of dormant cancer cell. Beyond its metabolic function, proline also influences other cellular processes such as epigenetic modifications and EMT^63,64^, raising the possibility that additional proline-mediated effects contribute to escape of DTCs from dormancy, representing a new avenue for future investigation.

In summary, our work reshapes the paradigm of tumor cell dormancy by demonstrating that intracellular collagen homeostasis and proline metabolism intersect to regulate NAD(H) balance, ultimately dictating dormancy or dormant cell awakening. These findings uncover new metabolic vulnerabilities that could be therapeutically exploited to eradicate dormant tumor cells before metastatic outgrowth occurs.

## MATERIAL AND METHODS

### Cell Culture

All cell lines used in this study are listed in Table 1 together with their proper culture media. The cells were maintained at 37C with 5% CO^2^ and were by checked free for mycoplasma contamination by PCR.

### Plasmids and siRNAs

A complete list of plasmids and siRNAs used in this study is provided in Table 2.

### Viral particles generation and cells infection

HEK293T were seeded at 80% confluency a day prior to transfection. Medium was changed on the day of transfection. Transfection was performed using Lipofectamin^™^ 2000 Transfection Reagent protocol (Thermo Fischer Cat #11668019). shRNAs in PLKO.1 lentiviral plasmid were encapsulated in lentiviral particles using the 3^rd^ generation system pVSVG/GAG-POL/TAT/REV.

Cells were then infected for 48hrs and selected with 5mg/ml of puromycin for at least 2 weeks. Knockdown and overexpression of P4HA2 were assessed by protein expression via western blot analysis.

### siRNA transfection

All siRNAs used in this study are listed in Table 2. Cells were seeded at 80% of confluency a day prior of transfection in 10cm dishes. The day of transfection medium was changed. 140pmol of siRNAs in OPTIMEM were used for transfection following the Lipofectamine^™^ RNAiMAX Transfection Reagent protocol (Thermo Fischer Cat #1377815).

At 48hrs post transfection cells were harvested for further experiments or used for inoculation in CAMs.

### Drug treatments and Proline supplementation

Prolyl-4-hydroxylase inhibitor (1,4 DPCA) inhibitor was purchased from Cayman Chemical (Cat#71220) and dissolved in DMSO. Cells were treated by adding 1,4 DPCA directly into the medium at different concentrations (1, 5,10mM) for 48hrs prior to being used for further analysis.

#### Proline In vitro supplementation:

L-Proline (Sigma Aldrich Cat# P0380) was dissolved in PBS and used fresh for each treatment. Cells were supplemented with L-Proline by adding it into the medium at the experimental defined concentration of 300mM for 48 hours prior to being used for further experiments^65^.

#### Proline In vivo supplementation:

L-Proline (Sigma Aldrich: Cat# P0380) was freshly dissolved in mice sterile drinking water (36g/L) and fed to the animals by replacing the solution every 2 days for a total of 19 days of supplementation^66^.

### Proline hydroxylation assay

To assess hydroxyproline levels we used the Hydroxyproline Assay kit (Colorimetric) Abcam (Cat#ab222941). All tissue lysates were prepared from freshly harvested tissues or snap-frozen samples. To homogenize the samples 100ml of dH_2_O was used for every 10 mg of tissue and thoroughly homogenize with an ultrasonic probe homogenizer. 100ml of 10N concentrated NaOH was added to 100ml of tissue homogenate and incubated at 120C for 1 hour.

Standard solutions were prepared all fresh at every use as recommended by the manufacture protocol. Measurements of hydroxyproline in each sample were made in triplicate and assessed using a standard curve reference as reported in the manufacture protocol.

### Western Blot analysis

Cells were homogenized on ice in RIPA buffer supplemented with cOmplete (Roche Cat#04693116001) and PhosSTOP (Roche Cat#04906837001) protease inhibitor cocktail tablets. Protein concentration was assessed with Pierce BCA protein kit from Thermo Scientific (Cat#23228). An albumin standard curve was used to assess protein concentration (Albumin Standard, Thermo Scientific Cat#23209) Western blot analysis was conducted using 50ug of total lysates on gels Antibodies used to probe the membranes are listed in Table 3. All primary antibodies were used at a dilution of 1:1000 in 5% bovine serum albumin (BSA) in 0.1% Tween-20 in TBS buffer. Signals were detected with HRP-conjugated secondary antibody and the chemiluminescence substrate SuperSignal^™^ West Pico PLUS from Thermo Scientific (Cat#34580). Equivalent loading was confirmed using b-actin antibody.

### Immunofluorescence in vitro

The day prior to the experiment cells were seeded on a coverslip and let adhere overnight.

After 12 hours, cells were washed with PBS buffer and fixed with 4% paraformaldehyde for 10 mins at room temperature. After fixation, cells were permeabilized with 0,2% Triton X-100 for 10 mins subsequently incubated for 1 hour with blocking buffer (3% BSA in PBS-Tween 0.1%, 10% Donkey serum) and then with primary antibodies listed in Table 3 diluted in blocking buffer.

Cells were washed 3 times with PSB buffer and incubated with the proper secondary antibody diluted in blocking buffer for 1 hour at room temperature. After secondary incubation, cells were washed 3 times with PBS buffer and incubated for 10 mins at room temperature with DAPI (Thermo Scientific Cat#62248) for nuclear staining. Coverslips were mounted on slides using Invitrogen^™^ Fluoromount-G^™^ polymerizing medium (Cat#00–4958-02) and images in a Leica SP5 confocal microscope.

For mitochondria ML analysis, rendering cells were imaged using Airyscan confocal LSM980 Zeiss microscope to collect z-stacks at high resolution. Imaris 10.0.1 (Oxford Instruments) was used to generate 3D rendered mitochondrial and nuclear objects as well as cell masks. The morphology and topology parameters^41^ (see Ref. 41) of 3D rendered mitochondrial objects were extracted from 20 cells from 5 z-stacks per cell condition. A custom-made Python script was used for the machine learning classification of the data exported from IMARIS. The algorithm utilized is a Random Forest Classifier provided by Python scikit learn 1.7. To reduce the rate of overfitting, 5 split stratified cross validation were done and the accuracy scores from each iteration averaged as the main accuracy score. The accuracy of the Random Forest algorithm is shown in two ways: i) confusion matrix which is a labeled table that compares the true label to the predicted label and shows the level of confusion of the classification model. ii) Proximity matrices show how close two objects are, based on how often they ended up in the same node of decision trees in the Random forest. The proximity matrix is graphed using a TSNE plot and the full code can be obtained at the following github link: https://github.com/barroso-lab/imarisextensions.git.

### Tissue Immunofluorescence

For tissue staining, 5μm sections were deparaffinized in xylene for 10 mins, treated with a graded series of alcohol (100%, twice/5mins, 95% twice/4mins, 70% twice/3mins), rehydrated in PBS, and washed in distilled water for 5 mins. For antigen retrieval tissue were treated with 10 mM citrate buffer (pH 6.0) for 20 mins on program HIGH of pressure cooker reference Cuisinart^®^ 6 qt. Electric Pressure Cooker. To permeabilize the cell membranes, sections were incubated in 0.1% Triton X-100 in PBS for 10 minutes at room temperature, a step that was omitted when the target of interest was primarily extracellular. This was followed by three additional washes in PBS. To minimize nonspecific binding, the sections were incubated in a blocking buffer consisting of 10% normal goat serum (NGS), 3% BSA and 0.1% Tween-20 in PBS for 1 hour at room temperature, with subsequent PBS washes.

For primary antibody labeling, antibodies were diluted in PBS containing 10% NGS and 0.1% Tween-20. Sections were incubated with the primary antibodies overnight at 4°C in a humidified chamber. A complete list of primary antibodies used in this study is provided in Table 3. Unbound primary antibodies were removed with three PBS washes, each lasting 5 minutes. Fluorophore-conjugated secondary antibodies were diluted in PBS containing 10% NGS, at a 1:500 dilution, and applied to the sections for one hour at room temperature in the dark.

Nuclei were counterstained with DAPI. Following secondary antibody incubation, sections were washed thoroughly with PBS and incubated in a DAPI solution (1:10000 in PBS) for 5 mins at room temperature. Three additional PBS washes were performed to remove excess DAPI.

Sections were mounted using the ProLong Gold anti-fade mounting medium. A coverslip was gently placed over the tissue to prevent air bubbles, preserving sections for imaging. Images were acquiered in a Leica SP5 confocal microscope.

### Multiplexed tissue immunofluorescence

Formalin-fixed paraffin-embedded xenograft tissue sections were prepared and iteratively stained as per Gerdes et al. (2013)^67^. Briefly, tissue sections were baked overnight at 60°C, deparaffinized with xylene, rehydrated by decreasing ethanol washes, followed by proprietary antigen retrieval optimized for multiple antigens (Leica Biosystems). Tissues were incubated in 0.3% Triton X-100 in PBS for 10 mins followed by blocking in 10% (w/v) donkey serum and 3% (w/v) bovine serum albumin (BSA) in PBS for 1 hour at room temperature. Primary antibodies were diluted in blocking solution and incubated for 1 hour at room temperature. A complete list of primary antibodies used is found in Table 3.

In-house primary antibody fluorescent conjugates were generated using the Alexa Fluor 647 labelling kit (Invitrogen, A20186) or the Alexa Fluor 555 labeling kit (Invitrogen, A20187). Nuclear labeling was performed using Hoechst (2 μg/ml, Invitrogen, H3570) for 10 mins at room temperature. Imaging was performed in 50% glycerol (v/v) in PBS. After imaging, dye inactivation was performed in an alkaline solution containing H_2_O_2_ for 15 mins with agitation followed by a wash in PBS buffer. Samples were reimaged following dye inactivation to measure residual fluorescence. Additional rounds of antibody staining and imaging were performed as described above, starting with primary antibody incubation. Image acquisition, flat-field correction, autofluorescence removal and registration were performed using the Cell DIVE Imager (Leica Biosystems). Single-cell segmentation was performed using Cellpose^68^ and intensity measurements were extracted using scikit-image (van der Walt et al. 2014). Host cells and non-proliferative regions were identified by positive staining of human-specific p53 and 53BP1 antibodies and a lack of phospho-H3 staining, respectively and were excluded from subsequent analysis. Measurements greater than 1.5x the interquartile range were identified as outliers and excluded from analysis.

### Rapid Autopsy samples

Patients who participate in the HCI Legacy to Life rapid autopsy program provide consent under the Total Cancer Care protocol (IRB approval #89989) and complete an autopsy authorization. Upon collection, tissues were defined as cancerous or grossly uninvolved by a pathologist or pathology assistant/diener. Formalin fixed paraffin embedded 5um sections of grossly uninvolved (GU) lung and liver tissues were prepared following the rapid autopsy of an HER2+ metastatic breast cancer patient. Immunostaining was performed as described below.

### Tumor tissue decellularization and extracellular matrix recolonization

Tissue decellularization: Tumors were extracted from the chorioallantoic membrane (CAM) of fertilized chicken eggs and carefully dissected to remove surrounding CAM tissue. Each tumor was placed in a 1.5 mL tube labeled according to the originally inoculated cell type. Decellularization was performed using a sequential buffer treatment. Tumors were first incubated in 1 mL of Decellularization Buffer 1 (10 mM Tris-HCl, pH 8.0, 0.1% EDTA, and 10 KIU/mL aprotinin) at 4°C under continuous shaking for 48 hours. The tissue was then transferred to a fresh 1.5 mL tube and incubated in 1 mL of Decellularization Buffer 2 (10 mM Tris-HCl, pH 8.0, 0.1% EDTA, 10 KIU/mL aprotinin, and 3% Triton X-100) under the same conditions for an additional 72 hours. Following the decellularization process, tumors were washed in PBS containing 2% penicillin-streptomycin (PBS + 2% PS) under sterile conditions in a tissue culture hood. Each tumor was then transferred to a fresh 1.5 mL tube containing 1 mL of freshly prepared enzymatic solution composed of 9.78 mL PBS + 2% PS, 20 μL RNase (1 mg/mL), and 200 μL DNase (10 mg/mL). Samples were incubated at 37°C for 24 hours. After enzymatic treatment, tumors were washed again in fresh PBS + 2% PS and returned to the incubator for an additional 24 hours. Same tissues were saved to be stained for DAPI (blue) to check the success of the decellularization procedure. At the end of the decellularization process, tumor extracellular matrices were ready for recolonization.

Recolonization: To recolonize the tumor ECM, 2.0 × 10^5 cells were cells (D-HEp3) were resuspended in 200 μL of culture medium and seeded onto each decellularized tumor scaffold. Samples were incubated at 37°C for several hours to allow cell attachment. At the end of the incubation period, the medium volume was increased to 500 μL per well with fresh culture medium, and tumors were returned to the incubator overnight. The following day, tumors were carefully transferred to new agar-coated wells and replenished with 500 μL of fresh culture medium. Samples were maintained in culture and incubated until the following Tuesday, at which point they were processed for imaging analysis.

### Metabolic flux analysis (Seahorse):

Metabolic analysis was performed using Seahorse XF96 Extracellular Flux Analyzer (Agilent Technologies). Key parameters of mitochondrial function were measured by using specific mitochondria respiration inhibitors (Seahorse XF Cell Mito Stress Test Kit, 103015–100, Agilent Technologies) while recording OCR values and following the manufacturer’s instructions. Briefly, prior to the start of the assay, cells were seeded onto a Seahorse XF96 Cell Culture Microplate (Agilent Technologies, 101085–004) at a density of 6×10^3^ cells per well. On the day of the assay, the medium was changed to assay media (DMEM glucose-free) supplemented with 25 mM glucose, 1 mM pyruvate, and 4 mM glutamine and incubated at 37°C without CO_2_ for 1h. Basal OCR following injection of different concentrations of oligomycin, carbonyl cyanide-4 (trifluoromethoxy) phenylhydrazone (FCCP), and rotenone were recorded 4 times. Running template was 3 min mix, 1 min wait, and 3 min measure. The results were normalized to the number of cells per well using CyQUANT^™^ Cell Proliferation Assay (Invitrogen, C7026) per the manufacturer’s instructions.

### NAD+/NADH assay

Cells were seeded and maintained in their growth media. Where applicable, cells were treated with 300 mM proline or subjected to P5CDH silencing induction for a period of 48 hours. Following the treatment or induction period, cells were harvested and subsequently stored as dry pellets at −80°C until further analysis. The NAD/NADH-Glo Assay (Promega, Cat. # G9071, G9072) was used to quantify total oxidized and reduced nicotinamide adenine dinucleotides (NAD+ and NADH) in biological samples. Frozen cells were lysed in a base solution containing 1% dodecyl trimethylammonium bromide (DTAB) to preserve dinucleotide stability. Lysed samples were split for separate NAD+ and NADH measurements. To measure NAD+, samples were treated with 0.4N HCl and heated at 60°C for 15 mins, while NADH samples were heated without acid treatment Both samples were then neutralized with Trizma base. An equal volume of NAD/NADH-Glo Detection Reagent was added to each sample, followed by gentle mixing and incubation at room temperature for 30–60 minutes. Luminescence was measured using a luminometer, with the light signal produced being proportional to the amount of NAD+ and NADH in the sample.

### Mass spectrometry analysis

Reagents and Chemicals: Ammonium hydroxide and ammonium carbonate were obtained from MilliporeSigma. LC-MS grade solvents, including water, methanol and acetonitrile, were purchased from Burdick and Jackson, Honeywell, Muskegon, MI (sourced through VWR). The stable isotope-labeled metabolites were obtained either from Cambridge Isotope Labs or Sigma Aldrich.

Sample Preparation: Cells were washed twice with PBS, harvested as dry pellets and frozen at −80 °C before extraction. Cell pellets were spiked with a mixture of nine stable isotope-labeled standards. A 500 μL aliquot of pre-chilled extraction solvent (80% methanol) was added to each sample to precipitate proteins. The samples were vortexed and incubated at −80 °C for 30 minutes. The protein was pelleted, and its concentration was used for normalization. The supernatant containing metabolites was dried, re-suspended in 20 μL of 80% methanol.

UHPLC-HRMS Metabolomics: Ultra-high performance liquid chromatography-high resolution mass spectrometry (UHPLC-HRMS) was performed using a Vanquish UHPLC interfaced with a Q Exactive HF quadrupole-orbital ion trap mass spectrometer (Thermo, San Jose, CA). Chromatographic separation was performed on an Atlantis Premier BEH Z-HILIC VanGuard FIT column (Waters, Milford, MA). The mobile phase was formed bt 10mM ammonium carbonate and 0.05% ammonium hydroxide in water (A), and 100% acetonitrile (B). The total running time was 20 minutes. The column temperature was set to 30°C, and the injection volume was 2 μL. The full MS was performed in positive and negative mode separately and the mass scan range is 65 to 950 m/z. Data analysis was performed using Xcalibur.

### Proteomics

#### S-trap protein digestion

Samples were homogenized in a buffer containing 100 ml of 5% SDS, 5mM DTT and 50mM triethylammonium bicarbonate, followed by 20mM iodoacetamide for 30 minutes in the dark. Afterward, phosphoric acid was added to the sample at a final concentration of 1.2%. Samples were diluted in six volumes of binding buffer (90% methanol and 10 mM ammonium bicarbonate, pH 8.0). After gentle mixing, the protein solution was loaded to an S-trap filter (Protifi) and spun at 500 g for 30 sec. The sample was washed twice with binding buffer. Finally, 1 μg of sequencing grade trypsin (Promega), diluted in 50 mM ammonium bicarbonate, was added into the S-trap filter and samples were digested at 37°C for 18 h. Peptides were eluted in three steps: (i) 40 μl of 50 mM ammonium bicarbonate, (ii) 40 μl of 0.1% TFA and (iii) 40 μl of 60% acetonitrile and 0.1% TFA. The peptide solution was pooled, spun at 1,000 g for 30 sec and dried in a vacuum centrifuge.

#### Sample desalting

Prior to mass spectrometry analysis, samples were desalted using a 96-well plate filter (Orochem) packed with 1 mg of Oasis HLB C-18 resin (Waters). Briefly, the samples were resuspended in 100 μl of 0.1% TFA and loaded onto the HLB resin, which was previously equilibrated using 100 μl of the same buffer. After washing with 100 μl of 0.1% TFA, the samples were eluted with a buffer containing 70 μl of 60% acetonitrile and 0.1% TFA and then dried in a vacuum centrifuge.

#### LC-MS/MS Acquisition and Analysis

Samples were resuspended in 10 μl of 0.1% TFA and loaded onto a Dionex RSLC Ultimate 300 (Thermo Scientific), coupled online with an Orbitrap Fusion Lumos (Thermo Scientific). Chromatographic separation was performed with a two-column system, consisting of a C-18 trap cartridge (300 μm ID, 5 mm length) and a picofrit analytical column (75 μm ID, 25 cm length) packed in-house with reversed-phase Repro-Sil Pur C18-AQ 3 μm resin. Peptides were separated using a 90 min gradient from 4–30% buffer B (buffer A: 0.1% formic acid, buffer B: 80% acetonitrile + 0.1% formic acid) at a flow rate of 300 nl/min. The mass spectrometer was set to acquire spectra in a data-dependent acquisition (DDA) mode. Briefly, the full MS scan was set to 300–1200 m/z in the orbitrap with a resolution of 120,000 (at 200 m/z) and an AGC target of 5×10e5. MS/MS was performed in the ion trap using the top speed mode (2 secs), an AGC target of 1×10e4 and an HCD collision energy of 35.

Proteome raw files were searched using Proteome Discoverer software (v2.5, Thermo Scientific) using SEQUEST search engine and the SwissProt human database. The search for total proteome included variable modification of N-terminal acetylation, and fixed modification of carbamidomethyl cysteine. Trypsin was specified as the digestive enzyme with up to 2 missed cleavages allowed. Mass tolerance was set to 10 pm for precursor ions and 0.2 Da for product ions. Peptide and protein false discovery rate was set to 1%. Following the search, data was processed as described by^69^. Briefly, proteins were log2 transformed, normalized by the average value of each sample and missing values were imputed using a normal distribution 2 standard deviations lower than the mean. Statistical regulation was assessed using heteroscedastic T-test (if p-value < 0.05). Data distribution was assumed to be normal but this was not formally tested.

### ECM Targeted Mass Spectrometry Imaging

FFPE tissues were deglycosylated by previously published protocols^70,71^ to increase access to ECM protein structure. In brief, these tissues were heated, deparaffinized, and antigen retrieved using 10 mM citraconic anhydride (pH 3) before PNGase F PRIME^™^ (N-zyme Scientifics) application (M5 HTX Technologies Sprayer) using 15 passes, 45 °C, 10 psi, 25 mL/min, 1200 velocity, 40 mm nozzle height, and a 2.5 mm offset. Tissues were placed in individual, preheated humidity chambers (37 °C) for a 2-hour enzymatic digestion, releasing N-glycans from the protein structure. Cleaved N-glycans and layer of PNGase F were washed off the tissue using 1 minute washes of ethanol, HPLC-grade water, high pH (10 mM tris, pH 9), and low pH (10 mM citraconic buffer, pH 3)^72^. A second antigen retrieval used 10 mM tris buffer (pH 9) for 25 minutes at 95 °C (vegetable steamer), further denaturing protein structure for protease digestion. Activity characterized collagenase type III (Worthington) was prepared at 0.01 mg/mL in 10 mM ammonium bicarbonate with 1 mM CaCl_2_. Automated spraying (HTX Technologies M5) employed 15 passes, 45 °C, 10 psi, 25 mL/min, 1200 velocity, 40 mm nozzle height, and 2.5 mm offset. This was followed by digestion in individual humidity chambers for 5 hours at 37 °C. After digestion, a-Cyano-4-hydroxycinnamic acid (CHCA) matrix (7 mg/mL in 50% acetonitrile and 1% TFA with Glu-1-Fibrinopeptide spiked in at 0.15 picomole) was sprayed by automated sprayer onto tissue using 10 passes, 79 °C, 10 psi, 70 mL/min, 1300 velocity, 40 mm nozzle height, and 3.0 mm offset. Slides were dried for > 30 minutes and then dipped once (<1 second) in cold 5 mM ammonium phosphate, monobasic and dried in a desiccator.

#### Mass Spectrometry Imaging

Tissues were imaged using Matrix-Assisted Laser Desorption/Ionization – Mass Spectrometry Imaging (MALDI-MSI) using a Bruker tims-TOF fleX in calibrated in positive ion mode. Sampling stepped a laser focused to 20 μm^2^ with 300 laser shots per pixel. Data from ECM peptides was collected over m/z range of 600–2500 using a transfer time of 75 μs and pre-pulse storages of 20 μs, respectively. MALDI-MSI data was imported into SCiLS Lab (Bruker) normalized to the internal standard for visualization. Maximum peak intensities from were exported for analyses. Peptides were used to perform image segmentation based on a K-means algorithm using Manhattan distance. Plots displayed were made in GraphPad software and analyses were done in MetaboAnalyst^73^.

#### Extracellular Matrix-Targeted LC-MS/MS Proteomics

After imaging experiments, 200 proof ethanol was used to remove CHCA matrix and the tissue scraped into a centrifuge tube. A total of 200 μL 25 mM ammonium bicarbonate/ 3 mM calcium chloride, pH: 7.4 was added to the tubes, and the tissues were briefly spun down using a benchtop centrifuge and sonicated to homogenize. Collagenase type III (5 μg) previously characterized by activity was added to the solution and sample digested overnight in a thermomixer (Fisher Scientific) Fiat 38 °C with 450 rpm shaking. Following overnight digestion, tissues were sonicated for 30 minutes, and a booster of characterized collagenase type III (2 μg) was added followed by 5-hour digestion in a thermomixer at 38 °C with 450 rpm shaking. The tissues were pelleted (10 mins at 14000 rpm) and the supernatant was collected and vacuum-concentrated until dry. A C18 Stagetip protocol (ThermoFisher; 10 μg capacity) was completed to remove intact enzyme. A C18 ZipTip, 2 μg capacity (ThermoFisher) was used to further ensure undigested proteins and salts were removed.

Pooled samples were injected in triplicate (200 ng) through a NanoElute coupled to a timsTOF FleX (Bruker Daltonics, Bremen Germany). Peptides were separated with a 40-minute gradient from 2% acetonitrile (0.1% formic acid) to 30% acetonitrile (0.1% formic acid) through an Aurora Ultimate ionopticks C18 column (75 μm inner diameter, 25 cm length, 1.7 μm particle size, 120 Å pore size). Peptides were eluted to a timsTOF FleX where data dependent acquisition was performed in positive ion mode with 150–1700 m/z mass range, 0.5–1.85 1/K0 trapped ion mobility range, 0.96 s cycle time with 8 PASEF ramps per cycle, 60 s transfer time, and 12 s pre pulse storage. Ion mobility and m/z were calibrated using a m/z = 622, 922, 1221 calibrant filter standard. System quality was assessed using Peirce^™^ HeLa Protein Digest Standard as a control at the beginning and end of the run.

Data was searched using Fragpipe v20.0 and MSFragger v3.8 using a nonspecific digest, searching for variable methionine oxidation, proline hydroxylation, and asparagine or glutamine deamidation with a protein and peptide false discovery rate of 0.01^74–82^. In MSFragger, fragment tolerance of 40 ppm and parent tolerance of 25 ppm were used. Data was searched against a curated SwissProt-reviewed database downloaded from UniProt on January 24, 2024, containing 1,020 entries and using search terms “homo sapiens” AND “extracellular matrix” OR “collagen” OR “elastin” OR “aggrecan” OR “gelatin” OR “osteonectin” OR “perlecan” OR “plasminogen” OR “fibronectin”. Reverse decoys and contaminants were added to the database using FragPipe v20.0 to help estimate false discovery rate and determine score cutoffs. Data were uploaded into Scaffold (v5.3.1) and a peptide probability of 99% was used to report peptide identifications. Quantitative values were normalized average precursor intensities exported from Scaffold. A hyperscore cutoff of >25 was used for matching peptides to imaging data.

### Two-photon imaging of tissues

Tissues were isolated from CAM and mice were imaged with an Olympus FVMPE-RS multiphoton laser scanning microscope using a XL Plan N 25x/1.05 W water immersion objective. Tissues were excited with a Insight X3 tunable ultrafast laser. For tissues expressing CDK2 sensor, excitation at 940nm (mVenus two photon excitation) was used followed by excitation at 880nm to acquiere SHG.

### Electron microscopy

Cells were fixed with 2% paraformaldehyde/ 2.5% glutaraldehyde in 0.1M sodium cacodylate solution (EMS, #15960–01) at 4C. Cells were rinsed in 0.1M sodium cacodylate buffer, then post fixed with 2% osmium tetroxide/1.5% potassium ferricyanide in 0.1M sodium cacodylate, and en bloc stained with aqueous 2% uranyl acetate. Cells were dehydrated in an ethanol series (25% up to 100%), infiltrated through an ascending ethanol/resin series and placed in resin overnight (EMS Embed 812 Kit, #14120). Samples were placed in a 60C vacuum oven for 72 hours to polymerize. Semithin sections (0.5 and 1 μm) were obtained using a Leica UCT ultramicrotome (Leica, Buffalo Grove, IL) and counterstained with 1% toluidine blue. Ultra-thin sections (80nms) were collected on Nickel 200 mesh grids (Ted Pella, Pelco, 7552N3) using a Coat-Quick adhesive pen (EMS, 70624). Sections were counter-stained with 1% uranyl acetate and lead citrate, imaged on an HT7500 transmission electron microscope (Hitachi High-Technologies, Tokyo, Japan) using an AMT NanoSprint12 12 megapixel CMOS TEM Camera (Advanced Microscopy Techniques, Danvers, MA).

#### Image analysis

All representative images shown in figures were chosen randomly from sets of images. Image analysis was performed with FIJI version ImageJ2 2.14.0/1.52p\f (National Institutes of Health; http://imageJ.nih.gov/ij) and IMARIS 10.0.

### In Vivo experiments

#### CAM Assay

Fertilized eggs sourced from Charles River were inoculated with 150,000 cells at embryonic day 10 of chicken development. Prior to inoculation, cells were detached using trypsin, and their concentration was determined using the Countess II (Invitrogen) cell counter with 0.4% Trypan blue stain to assess viability. Only viable cells were used to prepare a suspension in 1X D-PBS without calcium and magnesium.

To prepare the eggs, they were laid on their side, and the chorioallantoic membranes (CAMs) were dropped by puncturing the top and side of the eggshell (air sac side). Air was gently extracted from the air sac to create an air pocket above the CAM. A 50 μL volume of the prepared cell suspension was then introduced into the CAM using an insulin syringe (BD328468). After inoculation, the eggs were incubated at 37°C in a humidified environment for 6 days.

At embryonic day 16, the eggs were opened, and the tumors were carefully harvested. Tumor growth was assessed by mincing the tumors and digesting them for 20 mins at 37°C in a collagenase/BSA solution (2.5% BSA and 0.15% collagenase in PBS with magnesium and calcium, filtered) prepared from *Clostridium histolyticum* (Sigma Aldrich C0130). Tumor cell numbers were estimated by counting larger-diameter cells, distinguishing them from smaller host-derived chicken cells^35^.

#### Mice

##### Xenograft tumor model:

To ensure ethical compliance, tumor size did not exceed 1500 mm^3^. A total of 500,000 cells D-HEp3 shRNA control and 2 different D-HEp3 P4HA2 KD cells were injected orthotopically into the interscapular region of nude mice using sterile 1X DPBS. Each experimental group consisted of five mice. Tumor growth was monitored every two days by measuring the longest dimension with a Fisherbrand^™^ Traceable^™^ Digital Carbon Fiber Caliper. Tumor volume was determined using the formula V = 4/3πr3 and presented as individual values with SEM. Mice were humanely euthanized with CO_2_ if tumors reached a diameter of 1 cm. Any remaining animals were sacrificed on day 21. Tumors were preserved in paraffin for further histological analysis.

##### Lung metastasis model (Tail Vein injection):

For intravenous administration, tail vein injections were performed on nude mice under sterile conditions. Prior to injection, mice were warmed up using a heat lamp or placed on a warming pad for 5–10 minutes to dilate the tail veins. The injection site was cleaned with 70% ethanol to ensure proper visualization of the vein. A total of 100 μL of cell suspension (containing 5.0 × 10^5 cells in sterile 1X DPBS) was loaded into a 27G or 30G insulin syringe and injected into the lateral tail vein using a slow and steady technique to prevent vein rupture. Successful injection was confirmed by the absence of resistance and the lack of swelling at the injection site. If resistance was encountered or extravasation occurred, the injection was halted, and the mouse was monitored for any adverse effects.

After injection, mice were returned to their cages and observed for signs of distress or complications. Any animals displaying signs of pain or injection-related injury was excluded from the study following institutional ethical guidelines. Mice were humanely euthanized by cervical dislocation and lung tissues were collected for multiphoton imaging or preserved in paraffin for further histological analysis.

## STATISTICAL ANALYSIS

The investigators were not blinded to allocation during experiments and outcome assessment. Statistical methods were not employed to predetermine sample sizes, and no data were excluded from the analyses. Statistical analyses were performed with GraphPad PRISM (version 10.4.1 for MacOS, GraphPad Software, San Diego, California, USA, www.graphpad.com). Statistical tests used to analyze data are listed in the figure legends, and exact values of n and p values are either listed in the figure legends or displayed in figures. Results are expressed as mean ± SD. Differences were considered significant if p < 0.05 or lower. Sample size was determined empirically.

## Supplementary Material

**Supplemental Figure 1** A. Representative images of CAM nodules and tumors derived from D2.0R cells treated with DMSO (control) or with different concentrations (1,5,10 μM) of 1,4 DPCA. Nodules and tumors (n=5 CAMs per group) were harvested 6 days after inoculation and cells were counted per each tumor. Group comparisons were assessed using one-way ANOVA with Dunnett’s post-hoc test to identify differences relative to the control group.

B. Representative immunofluorescence images of P4HA2 (green), PDIA3 as an ER marker (red) and DAPI (blue) showing the specific endoplasmic reticulum localization and expression of P4HA2 (green) in D-HEp3 and T-HEp3 cells in CAMs. Scale bars: 10 μm.

C. Western blot analysis of P4HA2 protein levels in D-HEp-3 (dormant), R-HEp3 (re-awaken) and T-HEp-3 (proliferative) cells. b-actin was used as loading control.

D. Representative images of DTCs stained for P4HA2 (red) and the breast tumor marker HER2 (green) in grossly uninvolved (GU) lung and liver tissues collected from the rapid autopsy of a HER2+ metastatic breast cancer patient. DAPI was used for nuclei counterstaining. Scale bar: 20μm.

**Supplemental Figure 2** A. Western blot analysis for P4HA2 in D-HEp3 cells expressing siRNA control compared with D-HEp3 expressing 3 different siRNAs for P4HA2. β-actin as loading control.

B. Western blot analysis for P4HA2 in D-HEp3 cells expressing shRNA control compared with D-HEp3 expressing 3 different shRNAs for P4HA2. β-actin as loading control.

C: Western blot analysis for P4HA2 in D2.0R cells expressing control shRNA compared with D2.0R expressing 2 different shRNAs for P4HA2. β-actin as loading control.

D. Representative images of control D-HEp3 nodules and P4HA2 KD tumors from CAM assay after 6 days of inoculation. Lower panel: Number of cells in CAMs from D-HEp3 cells previously transiently transfected for 48 hours with siRNA Control or 3 different siRNAs for P4HA2. For each experimental group 12 CAMs were analyzed. For statistical comparison between groups was used the Dunnett’s test was used.

E. Percentage of PHH3 posite cells measured by immunofluorescence in control and P4HA2-depleted primary tumors in mice. n>10000 cells per condition from 3 different tumors.

F. Tumor volume measurements at 12 days after tumor inoculation for D-HEp3 shRNA Control and D-HEp3 shRNA P4HA2 injected orthotopically in the intrascapular space of athymic nude mice. Group comparisons were assessed using one-way ANOVA with Dunnett’s post-hoc test to identify differences relative to the control group.

G. Tumor latency analysis (from the experiment in F), defined as the time to reach a tumor volume of 100 mm^3^. Statistical significance was assessed using Dunnett’s multiple comparisons test.

H. Representative images of D2.0R control nodules and P4HA2 KD tumors in CAM. Graph represents number of cells from D2.0R control nodules (n=7 CAMs) and from D2.0R P4HA2 knockdown tumors (n=7 CAMs). The groups were statistically compared using Dunnett’s test.

I. Immunofluorescence images of single cells from D2.0R control and micrometastasis from P4HA2 knockdown cells in mice lungs. Scale bar: 50μm. Analysis of number of clusters per field (defined as a group of more than 20 cells) is shown. Data represent analysis from 5 mice per group (n=25 fields per condition).

J. Western blot analysis of P4HA2 protein levels in T-HEp3 control and P4HA2 overexpressing cells. β-actin as loading control.

K. Representative images of T-HEp3 control and P4HA2 overexpressing (OE) tumors in CAM. Graph represents number of cells from T-HEp3 tumors (n=11 CAMs) and from P4HA2 OE tumors (n=11 CAMs). The groups were statistically compared using Dunnett’s test.

**Supplemental Figure 3** A. Volcano plot analysis of differential gene expression between control and P4HA2 KD tumors in mice. Each point represents a gene plotted by log2(fold change) and log10(adjusted *p*-value). Red indicates significantly upregulated genes and blue indicates significantly downregulated genes (*adjusted p* < 0.05, log2FC > 1).

B. GO enrichment analysis of differentially expressed genes between P4HA2 KD and control orthotopic tumors, using WebGestalt (WEB-based Gene Set Analysis Toolkit, www.webgestalt.org. Bar plot displays significantly dysregualted biological process categories. Notably, extracellular matrix and collagen-containing extracellular matrix were among the most enriched terms, highlighting alterations in matrix-related pathways. Enrichment based on genes with adjusted *p* < 0.05.

C. Hematoxylin and eosin staining of D-HEp3 shRNA control nodules and D-HEp3 shRNA P4HA2-depleted awakened tumors used for MSI.

D. Image segmentation on 19-plex Collagen peptides. Heuristic spatial clustering produces localized ECM proteomes. Segmentation image analysis was produced from mass spectra data images by machine learning classification. Spectral colorized by (segmentation classification) localization within tissue. A legend of the cluster analysis is shown on the right.

E. Features ranked by their contributions to classification. Right: Pairwise score plot for top 5 components of the principal component analysis.

**Supplemental Figure 4** A. Representative immunofluorescence images and quantification for phospho-PERK (red) and DAPI as counterstaining for nuclei (blue) of D-HEp3 shRNA control compared with D-HEp-3 P4HA2 KD cells *in vivo* in CAM assays. Scale bar: 50μm.The graph shows the quantification of phospho-PERK intensity per cell (n= 60 cells were measured per group). Statistical analysis was performed using an unpaired two-tailed t-test.

B. Representative immunofluorescence images of PDIA3 (ER marker) in D-HEp-3 shRNA control cells and D-HEp-3 P4HA2 KD cells. Scale bar: 10μm. Quantification of PDIA3 staining intensity per cell (n=40 cells per group). Statistical analysis was conducted using an unpaired two-tailed t-test.

C. Western blot analysis panel of the different collagens (Col4A1, Col4A2, Col5A1, Col6A1 and Col12A1) in D-HEp3 shRNA control and P4HA2 KD cells upon treatment with ammonium chloride and leupeptin for 6 hours in control and P4HA2-depleted D-Hep3. Ponceau staining was used as loading control.

D. Representative images of LAMP1 (lysosomal markers) in red and COL5A1 (green) in control and P4HA2 KD cells. DAPI (blue) used as nuclei staining. Scale bar: 10μm.

E. Western blot analysis of COL5A1 protein levels upon proteasome inhibition (MG132 treatment for 6 hours) in D-HEp3 shRNA control cells compared to D-HEp-3 P4HA2 KD cells. Ponceau staining was used as a loading control.

E. Western blot analysis of COL5A1 in D-HEp3 cells control and upon transient transfection of small interfering RNA targeting COL5A1. Ponceau staining was used as a loading control.

**Supplemental Figure 5** A. Example of raw images and 3D-rendered processed Z-stack images collected via Airyscan high resolution imaging and used for ML pipeline for D-HEp3 TOM 20 and DAPI staining. 3D-rendered nuclei and mitochondria objects are shown.

B. Left: Plot shows the median of each cell line’s rendered mitochondria object number of single cells using TOM20 immunofluorescence staining, airyscan confocal microscopy and 3D rendering IMARIS image analysis software. Right: Similar analysis was used to plot the average volume per cell (μm^3^) of Tom20 mitochondrial 3D rendered objects.

C. ML Random Forest analysis based on topology (TPG) mitochondrial object parameters. Machine learning (ML) Random Forest algorithm shows high classification accuracy when using the topology parameter group (TPG, which includes the distance between mitochondrial objects and distance between mitochondrial and nucleus objects) comparing: top;D-HEp3 shRNA control, D-HEp3 P4HA2 shRNA2, D-HEp3 P4HA2 shRNA2 and T-Hep3; middle: D-HEp shRNA control, D-HEp3 P4HA2 shRNA2, D-HEp3 P4HA shRNA3 and bottom: T-HEp3, D-HEp3 P4HA2 shRNA2, D-HEp3 P4HA2 shRNA3. T-HEp3 cells are included as proliferative cell line to compare with P4HA2 KD cells. Confusion matrix and t-SNE plots show classification of these 4 cellular states using the Random Forest machine learning algorithm. Left: Confusion matrices based on the classification results of the Random Forest ML models of mitochondria for the TPG features. Each row corresponds to the true (known) label and column corresponds to the label predicted by the deep learning (DL) models. The main diagonal refers to correctly classified data points. The off-diagonal numbers represent incorrectly classified (misclassified) data points. (right panels). Right: T-SNE plots showing the high level of organization and clustering of the organelle objects per cell condition based on TPG features.

D. ML Random Forest analysis based on morphology (MPG) parameters. Machine learning (ML) Random Forest algorithm shows lower classification accuracy when using the morphology parameter group (MPG, which includes various mitochondrial object features related to their shape and size) comparing: Top: D-HEp3 shRNA control, D-HEp3 P4HA2shRNA2, D-HEp3 P4HA2 shRNA3 and T-Hep3; middle: D-HEp shRNA control, D-HEp3 P4HA2shRNA2, D-HEp3 P4HA2 shRNA3 and bottom: T-HEp3, D-HEp3 P4HA2shRNA2 and D-HEp3 P4HA2 shRNA3. T-Hep3 cells are included as proliferative cell line to compare with P4HA2 KD cells. Confusion matrix and t-SNE plots show classification of these 4 cellular states using the Random Forest machine learning algorithm. Left panels: Confusion matrices based on the classification results of the Random Forest ML models of mitochondria for the MPG features. Each row corresponds to the true (known) label and column corresponds to the label predicted by the deep learning (DL) models. The main diagonal refers to correctly classified data points. The off-diagonal numbers represent incorrectly classified (misclassified) data points. (right panels). Righ: T-SNE plots showing the reduced organization and clustering of the organelle objects per cell condition based on MPG features.

E. Confocal fluorescent images of D-HEp3 shRNA Control, P4HA2 shRNA1, and P4HA2 shRNA3 cells stably expressing mt-mKeima in basal conditions. Green fluorescence (488 nm), mitochondria. Red fluorescence (561 nm), mitophagy. Scale bar, 10 μm. Increased green/red colocalization indicates augmented mitophagic flux.

F. P4HA2 knockdown increases mitophagic flux in D-HEP3 cells. Quantification of the red (561 nm, acidic mitolysosomes)/green (488 nm, mitochondria) fluorescence ratio in mt-mKeima-expressing cells under basal conditions. P4HA2-depleted cells (shRNA1 and shRNA3) show a significant increase in the red/green ratio (Manders M1 colocalization) compared to shRNA Control, indicating enhanced mitophagic flux. Data represent mean ± SD of 20 cells per condition. Statistical significance was determined by one-way ANOVA with Tukey’s post hoc test.

**Supplemental Figure 6** A-B. Graphs show L-Glutamine and Succinate measurements in control CTR shRNA and P4HA2 KD cells (n=4). Group comparisons were assessed using one-way ANOVA with Dunnett’s post-hoc test to identify differences relative to the control group.

C. NAD+/NADH ratio (RLU: relative luminescence units) measurement in T-HEp3 (proliferative), R-HEp3 (re-awaken) and D-HEp3 (dormant) cell lines (n=4). Group comparisons were assessed using one-way ANOVA with Dunnett’s post-hoc test to identify differences relative to the control group.

D. NAD+/NADH ratio (as relative luminescence units RLU) measurement in T-HEp3 and D-HEp3 cells treated with Proline (Pro) for 48 hours or PBS used as vehicle. A one-way ANOVA was performed to evaluate differences among groups, with Dunnett’s test used post hoc to compare each treatment group to the control group (n=4).

E. Representative images of CAM nodules and tumors derived from D-HEp-3 shRNA control, D-HEp3 P4HA2 KD, D-HEp3 shRNA control with or without supplementation of proline for 48 hours and D-HEp3 shRNA transiently tranfected with P5CDH (ALDH4A1) siRNA with or without proline treatment. Tumors were harvested after 6 days after CAM inoculation. The bar graph shows the number of cells per nodules/tumor derived from each condition. Statistical analysis was performed using the Dunn’s multiple comparison test. (Number of CAMs analyzed: 9 D-HEp3 9 shRNA,P4HA2 shRNA, 9 D-HEp3 shRNA +Proline, 8 D-HEp3 9 shRNA +siP5CDH, 10 D-HEp3 9 shRNA +siP5CDH +Proline)

F. Upper panel: Scheme of the experimental design for *in vivo* lung metastasis assay with proline supplementation. Lower panel: Hematoxylin and eosin staining of lung sections from mice injected via tail vain with D-HEp3 shRNA control cells, proline supplemented D-HEp3 shRNA control cells, or D-HEp3 with P4HA2 KD cells. Scale bar: 5mm. The graph shows the number of lung metastases per mouse following tail vein injection of D-HEp3 P4HA2 KD and D-HEp3 shRNA control cells, with or without 300mM proline supplementation for 21 days. Group comparisons were performed using one-way ANOVA with Dunnett’s post-hoc test relative to the control group.

**Table 1.** List of cell lines

**Table 2.** List of shRNAs and siRNAs

**Table 3.** List of antibodies

Supplementary Files

This is a list of supplementary files associated with this preprint. Click to download.

- TABLE1.xlsx
- TABLE2.xlsx
- TABLE3.xlsx
- Suppl.Figure1.pdf
- Suppl.Figure2.pdf
- Suppl.Figure3.pdf
- Suppl.Figure4pdf.pdf
- Suppl.Figure5.pdf
- Suppl.Figure6.pdf
- Supplementallegends.docx

## Highlights

- P4HA2 links collagen hydroxylation to NAD(H) regulation and tumor dormancy
- Loss of P4HA2 triggers autophagy and increases mitochondrial respiration via ALDH4A1
- NAD+/NADH ratio is a metabolic switch controlling DTC dormancy and awakening
- Reactivated dormant cells rely on ALDH4A1 for survival, revealing a new therapeutic target for dormant cancer cells

## References

[1] Massagué J. & Ganesh K. Metastasis-Initiating Cells and Ecosystems. Cancer Discov. 11, 971–994 (2021). 33811127 10.1158/2159-8290.CD-21-0010 PMC8030695

[2] Sahai E. Illuminating the metastatic process. Nat. Rev. Cancer 7, 737–749 (2007). 17891189 10.1038/nrc2229

[3] Dupas A., Goetz J. G. & Osmani N. Extravasation of immune and tumor cells from an endothelial perspective. J. Cell Sci. 137, (2024). 10.1242/jcs.262066 39530179

[4] Goddard E. T. Immune evasion of dormant disseminated tumor cells is due to their scarcity and can be overcome by T cell immunotherapies. Cancer Cell 42, 119–134.e12 (2024). 38194912 10.1016/j.ccell.2023.12.011 PMC10864018

[5] Sosa M. S., Bragado P. & Aguirre-Ghiso J. A. Mechanisms of disseminated cancer cell dormancy: an awakening field. Nat. Rev. Cancer 14, 611–22 (2014). 25118602 10.1038/nrc3793 PMC4230700

[6] Bragado P., Sosa M. S., Keely P., Condeelis J. & Aguirre-Ghiso J. A. Microenvironments dictating tumor cell dormancy. Recent Results Cancer Res. (2012) doi:10.1007/978-3-642-28160-0_3. PMC3516301 22527492

[7] Phan T. G. & Croucher P. I. The dormant cancer cell life cycle. Nat. Rev. Cancer 20, 398–411 (2020). 32488200 10.1038/s41568-020-0263-0

[8] Dalla E., Sreekumar A., Aguirre-Ghiso J. A. & Chodosh L. A. Dormancy in Breast Cancer. Cold Spring Harb. Perspect. Med. 13, (2023). 10.1101/cshperspect.a041331 PMC10562521 37037598

[9] Risson E., Nobre A. R., Maguer-Satta V. & Aguirre-Ghiso J. A. The current paradigm and challenges ahead for the dormancy of disseminated tumor cells. Nat*. cancer* 1, 672–680 (2020). 10.1038/s43018-020-0088-5 PMC7929485 33681821

[10] Agudo J. Targeting cancer cell dormancy. Nat. Rev. Cancer 24, 97–104 (2024). 38062251 10.1038/s41568-023-00642-x PMC11038906

[11] Ganesh K. & Massagué J. Targeting metastatic cancer. Nat. Med. 27, 34–44 (2021). 33442008 10.1038/s41591-020-01195-4 PMC7895475

[12] De Martino D. & Bravo-Cordero J. J. Collagens in Cancer: Structural Regulators and Guardians of Cancer Progression. Cancer Res. 83, 1386–1392 (2023). 36638361 10.1158/0008-5472.CAN-22-2034 PMC10159947

[13] Mukherjee A. & Bravo-Cordero J. J. Regulation of dormancy during tumor dissemination: the role of the ECM. Cancer Metastasis Rev. 42, 99–112 (2023). 36802311 10.1007/s10555-023-10094-2 PMC10027413

[14] Sreekumar A. B3GALT6 promotes dormant breast cancer cell survival and recurrence by enabling heparan sulfate-mediated FGF signaling. Cancer Cell 42, 52–69.e7 (2024). 38065100 10.1016/j.ccell.2023.11.008 PMC10872305

[15] Di Martino J. S. A tumor-derived type III collagen-rich ECM niche regulates tumor cell dormancy. Nat. Cancer 3, 90–107 (2022). 35121989 10.1038/s43018-021-00291-9 PMC8818089

[16] Ohta Y. Cell-matrix interface regulates dormancy in human colon cancer stem cells. Nature 608, 784–794 (2022). 35798028 10.1038/s41586-022-05043-y

[17] Bragado P. TGF-β2 dictates disseminated tumour cell fate in target organs through TGF-β-RIII and p38α/β signalling. Nat. Cell Biol. (2013) doi:10.1038/ncb2861. PMC4006312 24161934

[18] Boyerinas B. Adhesion to osteopontin in the bone marrow niche regulates lymphoblastic leukemia cell dormancy. Blood (2013) doi:10.1182/blood-2012-12-475483. PMC3682335 23589674

[19] Wong M. Y. & Shoulders M. D. Targeting defective proteostasis in the collagenopathies. Curr. Opin. Chem. Biol. 50, 80–88 (2019). 31028939 10.1016/j.cbpa.2019.02.021 PMC6588406

[20] Myllyharju J. & Kivirikko K. I. Collagens, modifying enzymes and their mutations in humans, flies and worms. Trends Genet. 20, 33–43 (2004). 14698617 10.1016/j.tig.2003.11.004

[21] Myllyharju J. Prolyl 4-hydroxylases, the key enzymes of collagen biosynthesis. Matrix Biol. 22, 15–24 (2003). 12714038 10.1016/s0945-053x(03)00006-4

[22] Rappu P., Salo A. M., Myllyharju J. & Heino J. Role of prolyl hydroxylation in the molecular interactions of collagens. Essays Biochem. 63, 325–335 (2019). 31350381 10.1042/EBC20180053 PMC6744578

[23] Kim R. S. Dormancy signatures and metastasis in estrogen receptor positive and negative breast cancer. PLoS One (2012) doi:10.1371/journal.pone.0035569. PMC3329481 22530051

[24] Prockop D. J. Seminars in medicine of the Beth Israel Hospital, Boston. Mutations in collagen genes as a cause of connective-tissue diseases. N. Engl. J. Med. 326, 540–546 (1992). 1732793 10.1056/NEJM199202203260807

[25] Tschank G. Inhibition of prolyl hydroxylation and procollagen processing in chick-embryo calvaria by a derivative of pyridine-2,4-dicarboxylate. Characterization of the diethyl ester as a proinhibitor. Biochem. J. 275 (Pt 2, 469–476 (1991). 1850989 10.1042/bj2750469 PMC1150076

[26] Ishida Y. Autophagic elimination of misfolded procollagen aggregates in the endoplasmic reticulum as a means of cell protection. Mol. Biol. Cell 20, 2744–2754 (2009). 19357194 10.1091/mbc.E08-11-1092 PMC2688553

[27] Mizushima N., Levine B., Cuervo A. M. & Klionsky D. J. Autophagy fights disease through cellular self-digestion. Nature at 10.1038/nature06639 (2008). PMC2670399 18305538

[28] Lacombe A. & Scorrano L. The interplay between mitochondrial dynamics and autophagy: From a key homeostatic mechanism to a driver of pathology. Semin. Cell Dev. Biol. 161–162, 1–19 (2024). 10.1016/j.semcdb.2024.02.001 38430721

[29] Youle R. J. & Narendra D. P. Mechanisms of mitophagy. Nat. Rev. Mol. Cell Biol. 12, 9–14 (2011). 21179058 10.1038/nrm3028 PMC4780047

[30] Debnath J., Gammoh N. & Ryan K. M. Autophagy and autophagy-related pathways in cancer. Nat. Rev. Mol. Cell Biol. 24, 560–575 (2023). 36864290 10.1038/s41580-023-00585-z PMC9980873

[31] Dwyer S., Ruth J., Seidel H. E., Raz A. A. & Chodosh L. A. Autophagy is required for mammary tumor recurrence by promoting dormant tumor cell survival following therapy. Breast Cancer Res. 26, 143 (2024). 39425240 10.1186/s13058-024-01878-7 PMC11488247

[32] Vera-Ramirez L., Vodnala S. K., Nini R., Hunter K. W. & Green J. E. Autophagy promotes the survival of dormant breast cancer cells and metastatic tumour recurrence. Nat. Commun. (2018) doi:10.1038/s41467-018-04070-6. PMC5964069 29789598

[33] Franklin T. J., Morris W. P., Edwards P. N., Large M. S. & Stephenson R. Inhibition of prolyl 4-hydroxylase in vitro and in vivo by members of a novel series of phenanthrolinones. Biochem. J. 353, 333–338 (2001). 11139398 10.1042/0264-6021:3530333 PMC1221576

[34] Xiong G., Deng L., Zhu J., Rychahou P. G. & Xu R. Prolyl-4-hydroxylase α subunit 2 promotes breast cancer progression and metastasis by regulating collagen deposition. BMC Cancer 14, 1 (2014). 24383403 10.1186/1471-2407-14-1 PMC3880410

[35] De Martino D., Megino-Luque C. & Bravo-Cordero J. J. In Vivo Xenograft Model to Study Tumor Dormancy, Tumor Cell Dissemination and Metastasis. Methods Mol. Biol. 2811, 81–100 (2024). 39037651 10.1007/978-1-0716-3882-8_6 PMC11769578

[36] Spencer S. L. XThe proliferation-quiescence decision is controlled by a bifurcation in CDK2 activity at mitotic exit. Cell 155, 369–383 (2013). 24075009 10.1016/j.cell.2013.08.062 PMC4001917

[37] Ruth J. R. Cellular dormancy in minimal residual disease following targeted therapy. Breast Cancer Res. 23, 63 (2021). 34088357 10.1186/s13058-021-01416-9 PMC8178846

[38] Klionsky D. J., Cuervo A. M. & Seglen P. O. Methods for monitoring autophagy from yeast to human. Autophagy 3, 181–206 (2007). 17224625 10.4161/auto.3678

[39] Jin E. P4HA2 activates mTOR via hydroxylation and targeting P4HA2-mTOR inhibits lung adenocarcinoma cell growth. Oncogene 43, 1813–1823 (2024). 38654109 10.1038/s41388-024-03032-1 PMC11164680

[40] Senft D. & Ronai Z. A. UPR, autophagy, and mitochondria crosstalk underlies the ER stress response. Trends Biochem. Sci. 40, 141–148 (2015). 25656104 10.1016/j.tibs.2015.01.002 PMC4340752

[41] Wang L. Spatial topology of organelle is a new breast cancer cell classifier. iScience 107229, 10.1016/j.isci.2023.107229 (2023). PMC10384275 37519903

[42] Katayama H., Kogure T., Mizushima N., Yoshimori T. & Miyawaki A. A Sensitive and Quantitative Technique for Detecting Autophagic Events Based on Lysosomal Delivery. Chem. Biol. 18, 1042–1052 (2011). 21867919 10.1016/j.chembiol.2011.05.013

[43] Barbul A. Proline Precursors to Sustain Mammalian Collagen Synthesis12. J. Nutr. 138, 2021S–2024S (2008). 18806118 10.1093/jn/138.10.2021S

[44] Du J., Zhu S., Lim R. R. & Chao J. R. Proline metabolism and transport in retinal health and disease. Amino Acids 53, 1789–1806 (2021). 33871679 10.1007/s00726-021-02981-1 PMC8054134

[45] Sun M. G. Correlated three-dimensional light and electron microscopy reveals transformation of mitochondria during apoptosis. Nat. Cell Biol. 9, 1057–1065 (2007). 17721514 10.1038/ncb1630

[46] Olivares O. Collagen-derived proline promotes pancreatic ductal adenocarcinoma cell survival under nutrient limited conditions. Nat. Commun. 8, 16031 (2017). 28685754 10.1038/ncomms16031 PMC5504351

[47] Taylor H. Spatial Localization of Collagen Hydroxylated Proline Site Variation as an Ancestral Trait in the Breast Cancer Microenvironment. Matrix Biol. (2025) doi:10.1016/j.matbio.2025.01.006. PMC13356606 39863086

[48] Angel P. M. Zonal regulation of collagen-type proteins and posttranslational modifications in prostatic benign and cancer tissues by imaging mass spectrometry. Prostate 80, 1071–1086 (2020). 32687633 10.1002/pros.24031 PMC7857723

[49] Salmon H. Expansion and Activation of CD103+Dendritic Cell Progenitors at the Tumor Site Enhances Tumor Responses to Therapeutic PD-L1 and BRAF Inhibition. Immunity 44, 924–938 (2016). 27096321 10.1016/j.immuni.2016.03.012 PMC4980762

[50] Bonod-Bidaud C. & Ruggiero F. Inherited Connective Tissue Disorders of Collagens: Lessons from Targeted Mutagenesis. in (ed. Figurski D.) Ch. 13 (IntechOpen, Rijeka, 2013). doi:10.5772/50858.

[51] Ranganathan A. C., Adam A. P. & Aguirre-Ghiso J. A. Opposing roles of mitogenic and stress signaling pathways in the induction of cancer dormancy. Cell Cycle at 10.4161/cc.5.16.3109 (2006). PMC2517052 16929185

[52] Nagata K. HSP47 as a collagen-specific molecular chaperone: function and expression in normal mouse development. Semin. Cell Dev. Biol. 14, 275–282 (2003). 14986857 10.1016/j.semcdb.2003.09.020

[53] Lamandé S. R. & Bateman J. F. Procollagen folding and assembly: the role of endoplasmic reticulum enzymes and molecular chaperones. Semin. Cell Dev. Biol. 10, 455–464 (1999). 10597628 10.1006/scdb.1999.0317

[54] Liu G. Y. & Sabatini D. M. mTOR at the nexus of nutrition, growth, ageing and disease. Nat. Rev. Mol. Cell Biol. 21, 183–203 (2020). 31937935 10.1038/s41580-019-0199-y PMC7102936

[55] Werner E. & Werb Z. Integrins engage mitochondrial function for signal transduction by a mechanism dependent on Rho GTPases. J. Cell Biol. 158, 357–368 (2002). 12119354 10.1083/jcb.200111028 PMC2173123

[56] Irwin W. A. Mitochondrial dysfunction and apoptosis in myopathic mice with collagen VI deficiency. Nat. Genet. 35, 367–371 (2003). 14625552 10.1038/ng1270

[57] Parida P. K. Limiting mitochondrial plasticity by targeting DRP1 induces metabolic reprogramming and reduces breast cancer brain metastases. Nat. cancer 4, 893–907 (2023). 37248394 10.1038/s43018-023-00563-6 PMC11290463

[58] Otera H., Miyata N., Kuge O. & Mihara K. Drp1-dependent mitochondrial fission via MiD49/51 is essential for apoptotic cristae remodeling. J. Cell Biol. 212, 531–544 (2016). 26903540 10.1083/jcb.201508099 PMC4772499

[59] Cogliati S. Mitochondrial cristae shape determines respiratory chain supercomplexes assembly and respiratory efficiency. Cell 155, 160–171 (2013). 24055366 10.1016/j.cell.2013.08.032 PMC3790458

[60] Sessions D. T. Opa1 and Drp1 reciprocally regulate cristae morphology, ETC function, and NAD^+^ regeneration in KRas-mutant lung adenocarcinoma. Cell Rep. 41, (2022). 10.1016/j.celrep.2022.111818 PMC10265649 36516772

[61] Cui B. Pyrroline-5-carboxylate reductase 1 reprograms proline metabolism to drive breast cancer stemness under psychological stress. Cell Death Dis. 14, 682 (2023). 37845207 10.1038/s41419-023-06200-5 PMC10579265

[62] Choudhury D. Proline restores mitochondrial function and reverses aging hallmarks in senescent cells. Cell Rep. 43, 113738 (2024). 38354087 10.1016/j.celrep.2024.113738 PMC13092368

[63] D’Aniello C. Collagen Prolyl Hydroxylation-Dependent Metabolic Perturbation Governs Epigenetic Remodeling and Mesenchymal Transition in Pluripotent and Cancer Cells. Cancer Res. 79, 3235–3250 (2019). 31061065 10.1158/0008-5472.CAN-18-2070

[64] Comes S. L-Proline induces a mesenchymal-like invasive program in embryonic stem cells by remodeling H3K9 and H3K36 methylation. Stem cell reports 1, 307–321 (2013). 24319666 10.1016/j.stemcr.2013.09.001 PMC3849245

[65] Zhu J. Mitochondrial NADP(H) generation is essential for proline biosynthesis. Science 372, 968–972 (2021). 33888598 10.1126/science.abd5491 PMC8241437

[66] Mayneris-Perxachs J. Microbiota alterations in proline metabolism impact depression. Cell Metab. 34, 681–701.e10 (2022). 35508109 10.1016/j.cmet.2022.04.001

[67] Gerdes M. J. Highly multiplexed single-cell analysis of formalin-fixed, paraffin-embedded cancer tissue. Proc. Natl. Acad. Sci. U. S. A. 110, 11982–11987 (2013). 23818604 10.1073/pnas.1300136110 PMC3718135

[68] Pachitariu M. & Stringer C. Cellpose 2.0: how to train your own model. Nat. Methods 19, 1634–1641 (2022). 36344832 10.1038/s41592-022-01663-4 PMC9718665

[69] Aguilan J. T., Kulej K. & Sidoli S. Guide for protein fold change and p-value calculation for non-experts in proteomics. Mol. Omi. 16, 573–582 (2020). 10.1039/d0mo00087f 32968743

[70] Angel P. M. Mapping Extracellular Matrix Proteins in Formalin-Fixed, Paraffin-Embedded Tissues by MALDI Imaging Mass Spectrometry. J. Proteome Res. 17, 635–646 (2018). 29161047 10.1021/acs.jproteome.7b00713 PMC6637938

[71] Powers T. W. MALDI imaging mass spectrometry profiling of N-glycans in formalin-fixed paraffin embedded clinical tissue blocks and tissue microarrays. PLoS One 9, e106255 (2014). 25184632 10.1371/journal.pone.0106255 PMC4153616

[72] Clift C. L., Mehta A., Drake R. R. & Angel P. M. Multiplexed Imaging Mass Spectrometry of Histological Staining, N-Glycan and Extracellular Matrix from One Tissue Section: A Tool for Fibrosis Research. Methods Mol. Biol. 2350, 313–329 (2021). 34331294 10.1007/978-1-0716-1593-5_20

[73] Xia J. & Wishart D. S. Web-based inference of biological patterns, functions and pathways from metabolomic data using MetaboAnalyst. Nat. Protoc. 6, 743–760 (2011). 21637195 10.1038/nprot.2011.319

[74] da Veiga Leprevost F. Philosopher: a versatile toolkit for shotgun proteomics data analysis. Nature methods vol. 17 869–870 at 10.1038/s41592-020-0912-y (2020). 32669682 PMC7509848

[75] Venne K., Bonneil E., Eng K. & Thibault P. Improvement in peptide detection for proteomics analyses using NanoLC-MS and high-field asymmetry waveform ion mobility mass spectrometry. Anal. Chem. 77, 2176–2186 (2005). 15801752 10.1021/ac048410j

[76] Biniossek M. L. & Schilling O. Enhanced identification of peptides lacking basic residues by LC-ESI-MS/MS analysis of singly charged peptides. Proteomics 12, 1303–1309 (2012). 22589179 10.1002/pmic.201100569

[77] Geiszler D. J. PTM-Shepherd: Analysis and Summarization of Post-Translational and Chemical Modifications From Open Search Results. Mol. Cell. Proteomics 20, 100018 (2021). 33568339 10.1074/mcp.TIR120.002216 PMC7950090

[78] Kong A. T., Leprevost F. V, Avtonomov D. M., Mellacheruvu D. & Nesvizhskii A. I. MSFragger: ultrafast and comprehensive peptide identification in mass spectrometry-based proteomics. Nat. Methods 14, 513–520 (2017). 28394336 10.1038/nmeth.4256 PMC5409104

[79] Li K., Vaudel M., Zhang B., Ren Y. & Wen B. PDV: an integrative proteomics data viewer. Bioinformatics 35, 1249–1251 (2019). 30169737 10.1093/bioinformatics/bty770 PMC6821182

[80] Shteynberg D. D. PTMProphet: Fast and Accurate Mass Modification Localization for the Trans-Proteomic Pipeline. J. Proteome Res. 18, 4262–4272 (2019). 31290668 10.1021/acs.jproteome.9b00205 PMC6898736

[81] Teo G. C., Polasky D. A., Yu F. & Nesvizhskii A. I. Fast Deisotoping Algorithm and Its Implementation in the MSFragger Search Engine. J. Proteome Res. 20, 498–505 (2021). 33332123 10.1021/acs.jproteome.0c00544 PMC8864561

[82] Yu F. Fast Quantitative Analysis of timsTOF PASEF Data with MSFragger and IonQuant. Mol. Cell. Proteomics 19, 1575–1585 (2020). 32616513 10.1074/mcp.TIR120.002048 PMC7996969

